# A Quantitative Chemometric Study of Pharmaceutical Tablet Formulations Using Multi-Spectroscopic Fibre Optic Probes

**DOI:** 10.3390/ph17121659

**Published:** 2024-12-09

**Authors:** Peter J. G. Remoto, Keith C. Gordon, Sara J. Fraser-Miller

**Affiliations:** 1 The Dodd-Walls Centre for Photonic and Quantum Technologies, University of Otago, Dunedin 9016, New Zealand; rempe782@student.otago.ac.nz; 2 The Department of Chemistry, University of Otago, Dunedin 9016, New Zealand; 3 College of Science and Engineering, Flinders University, Bedford Park, South Australia 5042, Australia

**Keywords:** Raman, formulation, spatially-offset, fibre-optic

## Abstract

**Background/Objectives:** Two fibre optic probes were custom designed to perform Raman and near-infrared spectroscopic measurements. Our long-term objective is to develop a non-destructive tool able to collect data in hard-to-access locations for real-time analysis or diagnostic purposes. This study evaluated the quantitative performances of Probe A and Probe B using model pharmaceutical tablets. **Methods:** Measurements were performed using pharmaceutical tablets containing hydroxyl propylcellulose, titanium dioxide (anatase), lactose monohydrate, and indomethacin (γ form). Material content and thickness of bilayer samples (samples consisting of a top layer and a bottom layer of differing materials) were also assessed using Probe A to evaluate its capabilities to collect sub-surface information. Principal component analysis and partial least squares regression models were using individual and fused data to evaluate the performances of the different probe configurations. **Results:** Hydroxymethyl cellulose ($R_{P}^{2}=0.98$, RMSEP = 2.27% *w*/*w*) and lactose monohydrate ($R_{P}^{2}=0.97$, RMSEP = 2.96% *w*/*w*) content were most effectively estimated by near-infrared spectroscopy data collected using Probe A. Titanium dioxide ($R_{P}^{2}=0.99$, RMSEP = 0.21% *w*/*w*) content was most effectively estimated using a combination of 785 nm Raman spectroscopy and near-infrared spectroscopy using Probe B. Indomethacin ($R_{P}^{2}=0.97$, RMSEP = 1.01% *w*/*w*) was best estimated using a low-level fused dataset collected using 0 mm, 2.5 mm, and 5.0 mm lateral offsets of 785 nm spatially offset Raman spectroscopy using Probe A. **Conclusions:** The different probe configurations were able to reliably collect data and demonstrated robust quantitative performances. These results highlight the advantage of using multiple techniques for analysing different structures.

## 1. Introduction

Pharmaceutical products are typically composed of an active pharmaceutical ingredient (API) and excipients [1]. APIs are responsible for the therapeutic effect of the product, excipients take on many different roles, which include facilitating the formation and delivery of the API in an appropriate dosage form such as tablets, injectants, and disintegrants, to name a few [2,3,4,5]. Excipients can be used to modify the bioavailability of the API as they affect the solubility, stability, and dissolution rate of the product.

Hydroxypropyl methylcellulose (HPMC), titanium dioxide (TD), and lactose (LAC) are examples of commonly used pharmaceutical excipients that are used for various formulation purposes. HPMC and LAC, which belong to the polysaccharides and disaccharides families, respectively, are used as binding agent excipients in pharmaceutical formulations, to name one of their many uses. HPMC is a polysaccharide that is commercially available in different grades of viscosities. This can be used to tailor product stability and control drug release [6,7]. LAC belongs to the disaccharide family and is known to have two anhydrous forms (β-lactose and α-lactose) and a hydrate form (α-lactose monohydrate) [8,9,10]. TD is an inorganic substance frequently used as a coating agent because of its high refractive index, which protects light- or UV-sensitive APIs within the product from degradation [11]. Its well-known polymorphs are called rutile, brookite, and anatase [12]. Indomethacin (IND) is an active pharmaceutical ingredient used for analgesic and anti-inflammatory properties. It is used as the model API in this study. IND has eight known polymorphs (α, β, γ, δ, ε, ζ, η, and an 8th unnamed form) [13]. Different formulation approaches can impact the solid-state properties, particle size, and geometry of pharmaceutical products. This leads to the designing of various solid dosage forms, suspensions, and controlled-release formulations for different specific applications [14]. Moreover, hot melt extrusion, milling, and wet granulation can expose pharmaceutical ingredients to extreme environments which can create unintentional alteration of solid-state forms, and hence alter the physicochemical properties of the pharmaceutical products [15,16,17,18,19]. So, it is important that pharmaceutical manufacturers follow strict regulations and demonstrate compliance to ensure the output of consistently fit-for-purpose products.

Raman spectroscopy and near-infrared (NIR) spectroscopy are non-destructive techniques that collect information at a molecular level. These techniques can respectively probe certain molecular vibrations, which offer unique insights within chemical structures [16,20,21]. Coupled with appropriate fibre optics, such as those based on polymers and silicon glass, these spectroscopic techniques are able to be deployed in locations otherwise unreachable or hazardous using free space optics [22,23,24]. In-line implementation of these techniques for pharmaceutical applications has been extensively studied [25,26,27,28,29]. To name a notable few, Harting et al. demonstrated the use of the Raman spectroscopy via “PhAT probe” for continuous in-line API quantification during twin-screw wet granulation [30]. Bordos et al. performed in-line measurements using low-frequency Raman spectroscopy (also known as THz or low-wavenumber Raman spectroscopy) [31], a technique that collects lattice structure information of solids, to monitor the structural phase transitions of the API, such as paracetamol, from crystalline to amorphous as it is being dissolved in the polymer matrix. This information was then used to determine the solubility of the API with the changes in extrusion parameters. Saerens et al. evaluated the in-line NIR spectroscopic measurements in reflectance mode to non-destructively quantify API contents and used Raman spectroscopy to support their findings regarding drug-polymer hydrogen bond interactions [25,26].

**Figure 1:**
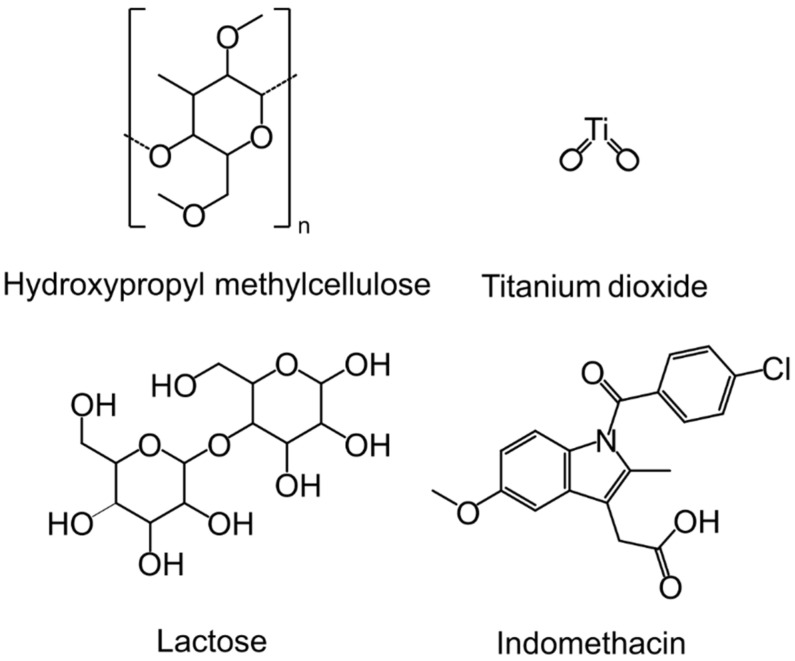
Molecular structures of hydroxypropyl methylcellulose, titanium dioxide, lactose, and indomethacin.

Our long-term objective is to develop a versatile multi-spectroscopic fibre optic probe with a wide range of uses where the real-time assessment or quality control using complementary spectroscopic data is of benefit, including diagnostic, food, and pharmaceutical applications [32,33]. In this study, the capabilities of two custom-made multi-spectroscopic fibre optic probe prototypes were investigated by using pharmaceutical tablets containing different ratios of hydroxypropyl methylcellulose (HPMC), α-lactose monohydrate (LAC-MH), anatase (a-TD), and γ-indomethacin (γ-IND) (Figure 1). Multivariate analysis data methods such as principal component analysis (PCA) and partial least squares regression (PLSR) were used to quantitatively and qualitatively evaluate their performance.

## 2. Results and Discussion

### 2.1. Raw Spectral Analysis

Raw Raman and NIR spectra of the tablet samples using the various configurations of Probe A and Probe B were presented in Figure S4. These tablet samples contain different ratios of HPMC, a-TD, LAC-MH, and γ-IND, which as a mixture of different chemical structures, displayed unique Raman and near-infrared spectra.

Raman spectroscopy measurements with 532 nm excitation were trialed due to the signal advantages associated with a shorter wavelength. However, emission (autofluorescence signals) was prominent in Raman spectra of the tablet samples with 532 nm excitation [34]. The relative intensity of which was material dependent because of their intrinsic excitation and relaxation properties. For example, the inorganic material, a-TD, exhibited strong Raman signals with little to no emissive components being detected. Whereas, γ-IND, which contains aromatic functional groups, showed relatively strong emission alongside its Raman signals. Raman spectra of the tablet samples irradiated with 785 nm light, on the other hand, presented little to no emission features. Electronic excited state transitions are dependent on the energy of light absorbed by the material. So, the emission can be varied by irradiating the material with a different wavelength of light. Raman spectra collected using Probe A, using either 532 or 785 nm light, presented strong signals at 670–3150 cm^−1^. These were attributed to the signal generated from the silicon glass of the fibre optic core. These strong silicon signals can obstruct weakly Raman active materials such as HPMC. This interference was addressed in the design of Probe B, where a Notch™ filter was installed at the tip of the probe which removed the silicon glass signals. However, this filter restricted Probe B’s access to the low-frequency Raman (LFR) region and the anti-Stokes region (−300 to −20, 20 to 300 cm^−1^). The LFR region is a particularly useful spectral window for pharmaceuticals as it contains low-energy vibrations that arise from the crystalline lattice (phonon modes) which can provide information regarding intermolecular interactions within a material [35]. Probe A was also designed to perform spatially offset Raman spectroscopy (SORS) [36,37]. The advantage of this design is that it can potentially probe useful sub-surface chemical information. However, the drawback of SORS is that an increasing offset between the point of excitation and collection reduces overall Raman signal intensity, and hence, increases the level of noise [38]. Probe B has an attachable optical accessory that enables side-on Raman and near-infrared measurements (i.e., parallel to the sample). This design offers an advantage in space-constrained environments where it prevents the probe from being perpendicular to the sample. However, as seen in Figure S4, this added optic led to a reduction in Raman signal intensity. Near-infrared spectra of Probe A and Probe B, as expected, present similar broad signals due to overtones and combination vibrational modes, which can be challenging to distinguish signals upon visual inspection. Given the intrinsic spectral features associated with each probe configuration, mathematical transformations of spectral datasets (known as spectral preprocessing) were essential to minimise noise and enhance useful information for further chemometric analysis, which were detailed in the previous section.

### 2.2. Qualitative Analysis

PCA is an unsupervised learning technique that reduces the dimensionality of a dataset to a few variables called principal components (PCs) composed of loadings and scores [39]. PC loadings show the contribution of each variable to the PC. PC scores are the projection of data onto the PC space. Each PC contributes to explaining the overall variance in the data. So, in the context of spectral analysis, PC loadings can reveal spectral information contained in the respective PC, whereas PC scores indicate how well much each spectrum in the dataset aligns with the spectral information contained in the respective PC.

**Figure 2:**
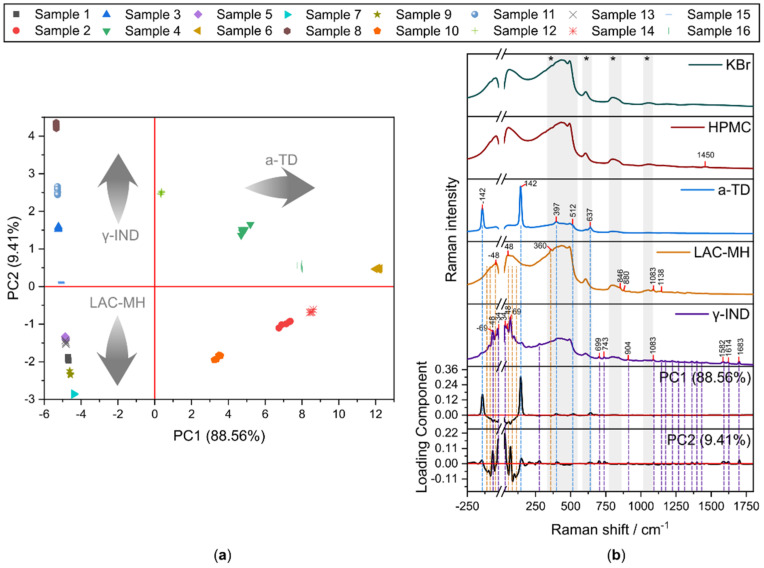
(**a**) Representative scores plot of first principal component (PC1) against second principal component (PC2) of A-785R-0 and (**b**) the corresponding loadings compared against reference spectra of KBr, HPMC, a-TD, LAC-MH, and γ-IND. Signal contributions from the silica glass are shaded grey and labeled with an asterisk (*).

PCA was separately performed on each probe configuration dataset. The first principal component (PC1) and the second principal component (PC2) explained > 70% of the variation for all the datasets. As can be expected, the PCs appeared to contain information regarding HPMC, a-TD, LAC-MH, and γ-IND according to their respective PC loadings (Figures S6–S16). In particular, PC1 and PC2 loadings of A-785R (Figure 2b) and A-532R (Figure S6) revealed spectral contributions that resembled a-TD, γ-IND, and LAC-MH Raman signals (see Figures S2 and S3) with minimal contributions from silicon glass signals. PC1 and PC2 loadings of B-785R showed Raman spectral contributions from HPMC, a-TD, γ-IND, and LAC-MH (see Figures S14 and S15). The appearance of HPMC signal contributions in the PC loadings showcased the utility of the Notch^TM^ filter to minimise silicon glass signal to reveal useful obscured information, such as weak HPMC Raman signals. For datasets involving NIR spectroscopy, their PC1 and PC2 loadings reveal that most spectral contributions come from HPMC and LAC-MH (see Figures S12, S15 and S16). These results demonstrate the capabilities of the probes to extract material-specific spectral information in complex mixtures, such as tablet samples. For A-NIR and B-NIR, their PC1 and PC2 loadings revealed spectral contributions from HPMC, LAC-MH, and γ-IND (Figures S4 and S6).

PC1 and PC2 scores plots of the probe configurations (Figure S5) clearly show the clustering of tablet samples, indicating that Probe A and Probe B output consistent Raman and NIR data. Moreover, the PC scores reflected the composition of the tablet samples and, hence, can be used to distinguish between tablet samples. PC1 and PC2 scores of A-532R-0 (Figure S5a) separated tablet samples according to their a-TD and γ-IND content. PC1 scores of A-NIR and B-NIR described and separated tablet samples according to their HPMC and LAC-MH content. A total of 785 nm Raman spectroscopy (using either Probe A or Probe B) was able to track the relative composition of the tablet samples more consistently than A-532R-0, such that a-TD and γ-IND were described by positive PC1 and positive PC2 scores, respectively. B-785R-F was able describe track relative HPMC content whereas A-785R-0 was able to track relative LAC-MH content, as described by their respective negative PC2 scores.

### 2.3. Quantitative Performance Analysis

PCA extracts PCs according to the variation within the dataset. Hence, PC loadings and scores do not give an accurate quantitative description of the tablet samples. Partial least squares (PLS), also known as a projection to latent space, is a supervised learning technique that develops latent variables which describe the maximised correlation between multiple datasets [40]. Here, the two pairs of datasets used were (1) the material of interest (HPMC, a-TD, LAC-MH, and γ-IND, separately) content dataset and (2) the probe configuration spectral dataset (both individually and using fused data approaches). PLSR training and prediction statistics such as coefficient of determination for calibration ($R_{C}^{2}$), validation ($R_{V}^{2}$), and prediction ($R_{P}^{2}$) and root mean squared error for calibration (RMSEC), validation (RMSEV), and prediction (RMSEP) for HPMC, a-TD, LAC-MH, and γ-IND were summarised in Table 1, Table 2, Table 3 and Table 4.

For both Probe A and Probe B, all configurations using NIR spectroscopy outperformed all configurations using Raman spectroscopy for predicting HPMC and LAC-MH (see Table 1 and Table 3, respectively). Specifically, A-NIR was the most effective dataset for predicting HPMC ($R_{P}^{2}=0.98$, RMSEP = 2.27% *w*/*w*) and LAC-MH ($R_{P}^{2}=0.97$, RMSEP = 2.96% *w*/*w*) content within the tablet samples. NIR light exhibits deeper sample penetration than visible light [41]. This allows for a larger sampling volume, which can lead to more accurate estimations, particularly for systems with depth such as tablets. Additionally, the structures of HPMC and LAC-MH show intrinsic sensitivity to NIR absorption. This is because of prominent vibrational modes associated with C-H and O-H, which are sigma-bonded in nature. These anharmonic vibrations, due to the involvement of the light hydrogen atom, allow for a greater spread of vibrational energy levels, which results in overtones and combination bands at lower energy levels [42]. Similarly, structures such as HPMC have weaker Raman scattering due to the lack of delocalised electrons in their structures [43]. NIR data regarding HPMC and LAC-MH was extracted by their respective PLS models shown in their regression coefficients in Figures S17 and S20. Extracted 532 nm autofluorescence (A-532B) was also explored and was found to be ineffective in quantifying material content within tablet samples, despite being an intrinsic property of material structure. The best performing model found was A-532B-2 predicting γ-IND content ($R_{P}^{2}=0.74$, RMSEP = 2.97% *w*/*w*). a-TD and γ-IND were most effectively estimated using data fusion approaches. Specifically, data-fused B-785R-F and B-NIR-F was the most effective dataset for predicting a-TD content ($R_{P}^{2}=0.99$, RMSEP = 0.21% *w*/*w*). A-532R-0 was a comparably effective dataset for predicting a-TD content ($R_{P}^{2}=0.96$, RMSEP = 0.58% *w*/*w*). This has demonstrated the utility of 532 nm excitation for Raman spectroscopy in effectively measuring inorganic materials with weak emission, such as those of a-TD [44], despite the presence of other materials with relatively strong emission. On the other hand, A-785R-0 ($R_{P}^{2}=0.97$, RMSEP = 1.07% *w*/*w*) outperformed A-532R-0 ($R_{P}^{2}=0.47$, RMSEP = 4.27% *w*/*w*) for quantifying γ-IND content, despite the efforts of minimising the obscuring effects of emission through fitting and subtraction. This has suggested that using 785 nm instead of 532 nm as an excitation source to avoid the spectral appearance of emission, albeit decreasing Raman scattering probability (i.e., decreased Raman signal intensity) [45], developed more effective quantitative models for materials that exhibit relatively strong emission. Furthermore, the A-785R-012 dataset (low-level fusion of A-785R-0, A-785R-1, and A-785R-2 data) was the most effective for predicting γ-IND content ($R_{P}^{2}=0.97$, RMSEP = 1.01% *w*/*w*) outperforming the individual techniques which comprised the fused dataset. However, the low-level fusion NIR dataset to the Raman datasets did not to contribute impactful improvements from the individual techniques. The regression coefficients of A-785R-012 for predicting γ-IND (Figure S21) and A-532R-0 for predicting a-TD (Figure S19) revealed that their low-frequency Raman signals had the largest contribution to their respective models. Some contributions from silicon glass signals were seen in the A-532R-0 PLS regression coefficients (Figure S18). Whereas Raman spectral contributions from materials were more apparent using B-785R-F. (Figure S19) These results have demonstrated the potential advantages of using a versatile tool capable of performing Raman and NIR spectroscopy to gather complementary information. In addition, the importance of spectral preprocessing to minimise spectral obstructions, such as those from silicon glass signals. Limitations of Raman spectroscopy can be covered by NIR spectroscopy, and vice versa. Therefore, this would be particularly useful for monitoring systems with diverse chemical species.

### 2.4. Bilayer Tablets

Bilayers were used to further assess the performance of Probe A for collecting sub-surface information. These bilayers were composed of a top layer tablet containing a mixture of HPMC and a-TD and a bottom layer composed of LAC-MH and γ-IND (see Table 5). PLSR output statistics were used to evaluate the overall performance of Probe A by quantifying HPMC, a-TD, LAC-MH, γ-IND, and estimating bilayer tablet thickness and were summarised in Tables S2–S6, respectively.

Apart from a-TD, the Probe A configurations quantified material content in bilayer samples (i.e., $R_{P}^{2}$ < 0.90) less effectively compared to tablet samples (see Tables S2–S5). Nevertheless, Probe A exhibited a level of performance in collecting sub-surface information from bilayers using SORS. For example, A-785R-0 was the most effective technique for quantifying materials which comprised the top layer, a-TD ($R_{P}^{2}$ = 0.96, RMSEP = 0.26% *w*/*w*) and HPMC ($R_{P}^{2}$ = 0.69, RMSEP = 3.78% *w*/*w*). For the bottom layer materials, LAC-MH was most effectively quantified by A-532R-0 ($R_{P}^{2}$ = 0.65, RMSEP = 5.75% *w*/*w*). Whereas γ-IND was the most effective dataset to quantify using A-532B-2 ($R_{P}^{2}$ = 0.89, RMSEP = 2.48% *w*/*w*) dataset. Regression coefficients for the A-785R-0 predicting a-TD revealed clear spectral contributions from a-TD with positive coefficients (see Figure S22). Regression coefficients for PLS models predicting HPMC (Figure S23) and LAC-MH (Figure S24) revealed barely discernible spectral contributions because of the associated noise. For example, regression coefficients for the A-785R-0 PLS model predicting HPMC revealed noisy and some barely distinguishable spectral contributions from LAC-MH and γ-IND with negative coefficients, indicating HPMC content in bilayers was tracked using diminishing LAC-MH and γ-IND Raman signals. Whereas regression coefficients of the A-532R-0 PLS model predicting LAC-MH content showed that the largest spectral contributions came from some LAC-MH Raman signals in the 2750–3150 cm^−1^ spectral region. These were interesting results because it was anticipated that A-785R would be more effective with quantifying bottom layer component content than A-532R because longer wavelengths were expected to travel deeper within the sample [45]. However, the top and bottom layers were separatable components (i.e., the top layer can replaced by another), which may have impacted how the light traveled within the bilayer. Moreover, these results have demonstrated the potential utility of extracted autofluorescence signals for quantifying materials such as γ-IND in the bilayer samples. However, the effectiveness of this technique to quantify γ-IND was not replicated by quantifying materials in the tablet samples.

Bilayer thickness estimation was also investigated using Probe A. However, most of the Probe A configurations were found to be ineffective, showing a negative coefficient of determination for prediction (see Table S6). The best performing technique for estimating thickness was A-785R-2 ($R_{P}^{2}$ = 0.31, RMSEP = 0.39% *w*/*w*) with its regression coefficients revealing noisy and barely discernible spectral feature contributions from LAC-MH and γ-IND with negative coefficients.

## 3. Materials and Methods

### 3.1. Materials

Commercial hydroxypropyl methylcellulose (>90–<100%, Food grade) was purchased from the DOW Chemical Company (Midland, MI, USA). Commercial titanium dioxide (M_w_ = 71.87 g mol^−1^, 99.7%) was purchased from Sigma-Aldrich (St. Louis, MI, USA). Commercial lactose (monohydrate, M_w_ = 360.32 g mol^−1^, Lab grade) was purchased from Unilab (Ajax Laboratory Chemicals, Wollongong, NSW, Australia). Commercial indomethacin (≥99%, TLC grade) was purchased from Sigma-Aldrich, inc. (St. Louis, MI, USA). Chemical structures of these materials are presented in Figure 1.

### 3.2. Preparation of Pharmaceutical Tablets

#### Pharmaceutical Mixtures

A total of 200 mg powder pre-mixes were made by mixing hydroxypropyl methylcellulose, titanium dioxide, lactose, and indomethacin. The pre-mixes were compressed using an 8 mm stainless steel die, punches, and a hydraulic press (PerkinElmer Inc., Waltham, MA, USA) with 2.5 kPa for 60 s. A total of 16 individual tablets were prepared with each sample possessing a unique composition. The compositions of these tablet samples are presented in Table 6.

Psuedo-bilayers (BL) were prepared by placing two layers on top of each other. The bottom layer is composed of lactose and indomethacin, while the top layer is composed of hydroxymethyl cellulose and titanium dioxide. The total compositions of these tablet samples are presented in Table 5.

The compositions of the pharmaceutical tablets and the BL were chosen to cover a broad range of pharmaceutical ingredient ratios, which ensured a diverse spread of values. Additionally, structural integrity (i.e., no cracks or crumbling) was observed with these compositions, making the tablets suitable for measurements.

### 3.3. Spectroscopic Methods

#### 3.3.1. Raman Spectroscopy

Raman spectroscopy measurements were performed on in-house built free space setups (see Figure S1–S3). These are labeled Raman Setup 1, Raman Setup 2, and Raman Setup 3.

Raman Setup 1 used a Sapphire 532-100 SF NX CDRX laser (Coherent, Germany) with a 50 μm entrance slit to irradiate the sample with 532 nm excitation. The backscattered light was collected using a collimating lens and then dispersed horizontally by a 600-groove mm^−1^ grating onto a liquid nitrogen cooled (153 K) Isoplane and PyLON 400 BR CCD (Princeton Instruments, Trenton, NJ, USA). Data measurements were performed across a spectral window −677 to 3174 cm^−1^ with 1 s exposure with 60 co-accumulations, controlled using WinSpec/32 software (Roper Scientific, Ottobrunn, Germany). Sulfur, 1,4 bis(2-methylstyryl) benzene, and a 1:1 toluene and acetonitrile mixture were used as standards to calibrate the CCD pixels.

Raman Setup 2 is composed of a 785 nm laser module (Ondax Inc., Morovia, CA, USA) as an excitation source and its spontaneous emission from the laser module was removed via BragGate bandpass filter (OptiGrate Corp., Oviedo, FL, USA) and focused into an LS 785 spectrograph (Princeton Instruments, Tenton, NJ, USA) via fibre optic cables which dispersed Raman scattered light onto a Pixis 100 BR CCD (Princeton Instruments, Trenton, NJ, USA) and controlled using Winspec/32 software (Roper Scientific, Ottobrunn, Germany). The measurements were collected in the spectral window −359 to 2040 cm^−1^ with 1 s exposure time and 60 co-accumulations. Sulfur and 1,4 bis(2-methylstyryl) benzene were used as standards to calibrate the CCD pixels.

Raman Setup 3 used a 785 nm laser module (Ondax Inc., Morovia, CA, USA) with a 50 μm entrance slit to irradiate the sample with 785 nm excitation. The backscattered light was directed via fibre optics to a 300-groove mm^−1^ grating and was dispersed horizontally onto a liquid nitrogen-cooled (153 K) Isoplane and PyLON 400 BR CCD (Princeton Instruments, Trenton, NJ, USA). Data measurements were performed across a spectral window −250 to 3258 cm^−1^ with 1 s exposure with 60 co-accumulations, controlled using WinSpec/32 software version 2.5 (Roper Scientific, Ottobrunn, Germany). Sulfur, 1,4 bis(2-methylstyryl) benzene, and a 1:1 toluene and acetonitrile mixture were used as standards to calibrate the CCD pixels.

#### 3.3.2. Near-Infrared (NIR) Spectroscopy

Reflectance near-infrared spectroscopy measurements were performed using a broadband (360–2600 nm) tungsten-halogen light source (Thorlabs, Newton, NJ, USA) and a WP NIR I spectrometer (Wasatch Photonics, Morrisville, NC, USA) equipped with a TEC-cooled G9214-512S InGaAs array (−15 °C ± 1 °C). This was controlled using the Enlighten Spectroscopy software version 4.1.6 (Wasatch Photonics, NC, USA). Measurements were collected using 10 ms and 60 scans per spectrum with a 3 mm working distance from the sample. Dark and background reference spectra were collected at the start of the experiment. A polytetrafluoroethylene (PTFE) reflectance standard was used for the background reference.

### 3.4. Fibre Optic Probes

The spectroscopic measurement setups were coupled to multi-spectroscopic fibre optics designed to transmit light with a wavelength range of 400–2400 nm. These fibres are equipped with a Technology Enhanced Clad Silica (TECS) multi-mode fibre (Thorlabs Inc., Newton, NJ, USA) with low hydroxyl content. This fibre, is composed of a pure silica core (200 ± 5 μm diameter), TECS hard cladding (225 ± 5 μm diameter), and tefzel coating (500 ± 5 μm diameter). Two designs of multi-spectroscopic probes were used in this study:

(1) Probe A was made in-house and features three adjacent probe tips (~2.5 mm apart), all of which contained near-infrared source and detector fibre lines. However, only the first probe tip contains a Raman source as well as a detector line, while the other two only contain Raman detector fibre lines and thus design to take spatially offset measurements. This design also had no filtering at the probe tip to allow for low wavenumber measurements and different λ’s.

(2) Probe B features a compact probe with Raman, near-infrared (NIR), and optical coherence tomography (not evaluated in this study) source and detection lines. This probe has a notch filter at the tip of the Raman collection line and was custom made by EMvision LLC (Loxahatchee, FL, USA) with forward and side facing measurement configurations.

A description of each probe configuration can be found in Table S1.

### 3.5. Spectral Preprocessing

All Raman spectra were subjected to cosmic spike removal with a width of five pixels and subsequently converted from SPE to XLS file format using Spectragryph software version 1.2.17 (Obserstdorf, Germany) [46]. Care was taken during spectral preprocessing to emphasise useful information (e.g., signals from pharmaceuticals) and minimise non-useful signals (e.g., baseline components, silicon glass signals, and artefacts) by inspecting the spectra for each preprocessing step and inspecting the latent variables developed during multivariate analysis.

#### 3.5.1. Raman Setup 1 (Raman Spectroscopy via Probe A with 532 nm Excitation)

An emissive baseline component (i.e., autofluorescence band) was observed at 670 to 3150 cm^−1^ for samples subjected to 532 nm excitation. This spectral range was therefore fitted and subtracted with a seventh-order polynomial using OriginPro (OriginLab Croporation, Northampton, MA, USA). A linear baseline correction at −250 to 250 cm^−1^ using ModPoly baseline algorithm with first-order polynomial from the PyBaseline library [47]. This was completed to conserve information within the vibrational density of states (VDOS). A baseline offset was applied at 250 to 670 cm^−1^ to minimise the impact of the silicon glass signal on the development of latent variables during multivariate analysis. These baseline corrections were followed by individually applying standard normal variate (SNV) to the following spectral windows: (1) −250 to −20, 20 to 250 cm^−1^; (2) 250 to 1800 cm^−1^; and (3) 2750 to 3150 cm^−1^. This ensured that the low-, mid-, and high-wavenumber regions have similar intensity scales to have similar contributions during multivariate analysis.

#### 3.5.2. Raman Setup 2 (Raman Spectroscopy via Probe A with 785 nm Light)

Linear baseline corrections were individually applied at −250 to 250 cm^−1^ and 670 to 1800 cm^−1^ using the ModPoly baseline algorithm with first-order polynomial from the PyBaseline library [47]. A baseline offset correction was applied at the range of 250 to 670 cm^−1^. These baseline corrections were followed by individually applying SNV to the following spectral windows: (1) −250 to −20, 20 to 250 cm^−1^; and (2) 250 to 1800 cm^−1^. This ensured that the low- and mid-wavenumber regions have similar intensity scales to have similar contributions during multivariate analysis.

#### 3.5.3. Raman Setup 3 (Raman Spectroscopy via Probe B with 785 nm Light)

A baseline, which originated from the make of Probe B, was observed at 320 to 3258 cm^−1^. Asymmetric least squares (AsLS) smoothing (λ = 1,000,000, *p* = 0.01, 10 iterations) was used to individually fit and subtract the baseline at 330 to 1800 cm^−1^ and at 2750 to 3150 cm^−1^, followed by SNV.

#### 3.5.4. Near-Infrared Spectroscopy

The same preprocessing steps were applied to the NIR spectroscopy data collected via Probe A and Probe B but with the spectral windows adjusted according to the noise observed in the respective NIR spectra. For Probe A, SNV followed by Savitsky–Golay filter (second-order polynomial, second-order derivative, with 13-point window) were applied to 915 to 1730 nm using SciPy version 1.11.1 [48]. For Probe B, these steps were applied to 900 to 1745 nm.

### 3.6. Low-Level Data Fusion

Data fusion was performed to combine complementary information from both techniques. This can potentially lead to developing latent variables more effectively than from individual datasets. Initially, the preprocessed Raman and NIR spectra had different intensity scales due to the nature of the preprocessing steps outlined in the previous section. Therefore, preprocessed NIR spectra were scaled up 1000-fold to align the intensity scales of the two datasets (NIR and Raman). Preprocessed Raman and scaled preprocessed NIR data collected through the same probe (Probe A or Probe B) were concatenated to create a low-level fused dataset. The number of x-variables (e.g., wavelength or wavenumbers) was not considered because these represent the nature of each respective spectra (e.g., broad NIR peaks in contrast to sharp Raman signals). Hence, the focus was on the scaling.

### 3.7. Multivariate Analysis

#### 3.7.1. Principal Component Analysis (PCA)

All preprocessed data (both model set and test set) were used to fit the PCA model with singular value decomposition using the Scikit-Learn (version 1.3.0) library [49].

#### 3.7.2. Partial Least Squares Regression (PLSR) 

PLSR models were trained using the model set data (see Table 6) to estimate HPMC, a-TD, LAC-MH, and γ-IND content *w*/*w* % using the PLSRegression module with the non-iterative partial least square (NIPALS) algorithm from the Scikit-Learn (version 1.3.0) library [49]. Additionally, 16-fold cross validation was performed to ensure that the model is not overfit. PLSR models with 18-fold cross-validation were trained using a bilayer sample model set (see Table 5) for bilayer samples with the pharmaceutical ingredients and sample thickness. The PLSR models were evaluated by estimating the test set data using the coefficient of determination (R^2^) and root mean square error (RMSE) as metrics.

## 4. Conclusions

Two custom-made multi-spectroscopic fibre optic probes were developed with two different designs. Both Probe A and Probe B could enable the collection of Raman and near-infrared data through fibre optics. Probe A has multiple collection lines which allow the collection of spatially offset Raman spectroscopy. This study investigated their capabilities to consistently and reliably collect Raman and near-infrared spectroscopy data using tablet and pseudo-bilayer formulations. Overall, this study has shown that the use of Raman and NIR spectroscopies have their own distinct advantages and limitations in detecting different chemical information. Moreover, low-level fusion (i.e., concatenation of preprocessed data) was found to be an effective approach to generally improve quantitative performance for the select materials. So, the collection of both Raman and NIR data is of particular value, especially in the context of developing tools capable of collecting data from relatively inaccessible locations, such as that of a gastrointestinal tract [33], where a diverse species of biological materials can exist. Further developments of multi-spectroscopic tools, which take advantage of complimentary techniques, can expand the horizons for vibrational spectroscopy to be used for further used for non-destructive diagnostics, product quality control, and real-time analysis.

## Figures and Tables

**Table 1:** Quantitative performance of predicting HPMC content in tablet samples. The PLS was created for HPMC in the range of 0 to 100% *w*/*w*. ***Bold and italic text*** indicates the most effective model for quantifying HPMC.

Technique	Number of Factors	$R_{C}^{2}$	RMSEC/% *w*/*w*	$R_{V}^{2}$	RMSEV/% *w*/*w*	$R_{P}^{2}$	RMSEP/% *w*/*w*
A-532R-0	4	0.98	5.00	0.83	13.47	0.88	5.59
A-532R-1	4	0.98	4.51	0.73	17.26	0.78	7.66
A-532R-2	2	0.87	12.04	−0.41	39.24	−0.70	21.33
A-532B-0	2	0.49	23.45	0.04	32.28	−0.94	22.82
A-532B-1	3	0.78	15.42	0.25	28.51	−0.88	22.43
A-532B-2	1	0.43	24.90	0.24	28.85	−0.95	22.90
A-785R-0	4	0.98	4.90	0.92	9.19	0.91	4.88
A-785R-1	4	0.98	4.74	0.92	9.21	0.86	6.11
A-785R-2	4	0.98	5.09	0.87	11.88	0.59	10.49
***A-NIR***	***3***	***0.99***	***2.36***	***0.98***	***4.64***	***0.98***	***2.27***
B-785R-F	3	0.97	6.17	0.86	12.19	0.77	7.89
B-785R-S	3	0.97	5.96	0.87	12.00	0.63	9.96
B-NIR-F	2	0.95	7.26	0.70	18.18	0.96	3.39
B-NIR-S	3	0.99	2.43	0.98	5.10	0.97	3.02
A-532R-012	5	0.99	2.36	0.66	19.22	0.24	14.25
A-532R-0+NIR	1	0.88	11.25	0.80	14.71	0.89	5.55
A-532R-1+NIR	2	0.92	9.09	0.79	15.01	0.88	5.57
A-532R-2+NIR	2	0.98	4.92	0.87	12.05	0.95	3.67
A-532R-012+NIR	2	0.91	9.71	0.78	15.31	0.81	7.07
A-785R-012	3	0.97	5.76	0.86	12.36	0.79	7.52
A-785R-0+NIR	3	0.98	4.12	0.91	9.83	0.93	4.22
A-785R-1+NIR	3	0.98	4.16	0.90	10.63	0.94	3.92
A-785R-2+NIR	3	0.99	3.70	0.92	9.32	0.95	3.64
A-785R-012+NIR	3	0.98	4.47	0.85	12.97	0.95	3.50
B-785R-F+NIR	2	0.93	8.91	0.79	15.23	0.80	7.29
B-785R-S+NIR	3	0.98	5.19	0.88	11.39	0.90	5.13

RMSEC = root mean squared error of calibration; RMSEV = root mean squared error of validation; RMSEP = root mean squared error of prediction.

**Table 2:** Quantitative performance of predicting a-TD content in tablet samples. The PLS was created for a-TD in the range of 0 to 10.2% *w*/*w*. ***Bold and italic text*** indicates the most effective fused model for quantifying a-TD.

Technique	Number of Factors	$R_{C}^{2}$	RMSEC/% *w*/*w*	$R_{V}^{2}$	RMSEV/% *w*/*w*	$R_{P}^{2}$	RMSEP/% *w*/*w*
A-532R-0	1	0.85	1.34	0.66	2.02	0.96	0.58
A-532R-1	1	0.91	1.03	0.82	1.47	0.71	1.56
A-532R-2	1	0.89	1.15	0.59	2.21	0.37	2.30
A-532B-0	5	0.66	2.02	−0.72	4.55	0.45	2.16
A-532B-1	5	0.59	2.21	−0.08	3.60	0.61	1.81
A-532B-2	5	0.36	2.77	−0.16	3.73	0.35	2.33
A-785R-0	1	0.99	0.28	0.98	0.47	0.83	1.18
A-785R-1	1	0.99	0.35	0.97	0.62	0.63	1.77
A-785R-2	1	0.99	0.26	0.99	0.32	0.81	1.27
A-NIR	2	0.46	2.55	−0.64	4.44	0.64	1.74
B-785R-F	1	0.92	0.95	0.83	1.43	0.96	0.56
B-785R-S	1	0.93	0.90	0.84	1.40	0.96	0.58
B-NIR-F	3	0.82	1.47	−0.19	3.79	0.69	1.63
B-NIR-S	2	0.58	2.25	−0.37	4.05	0.69	1.61
A-532R-012	1	0.92	0.96	0.79	1.58	0.90	0.90
A-532R-0+NIR	2	0.91	0.99	0.76	1.70	0.97	0.54
A-532R-1+NIR	1	0.76	1.70	0.54	2.36	0.86	1.07
A-532R-2+NIR	1	0.97	0.62	0.56	2.30	0.53	2.00
A-532R-012+NIR	1	0.91	1.07	0.71	1.87	0.95	0.65
A-785R-012	1	0.99	0.25	0.98	0.45	0.75	1.44
A-785R-0+NIR	2	0.99	0.24	0.97	0.59	0.86	1.10
A-785R-1+NIR	1	0.82	1.45	0.68	1.95	0.91	0.88
A-785R-2+NIR	1	0.99	0.26	0.98	0.47	0.81	1.25
A-785R-012+NIR	1	0.95	0.77	0.90	1.09	0.90	0.91
***B-785R-F+NIR***	***1***	***0.93***	***0.93***	***0.79***	***1.59***	***0.99***	***0.21***
B-785R-S+NIR	1	0.93	0.88	0.81	1.51	0.99	0.32

RMSEC = root mean squared error of calibration; RMSEV = root mean squared error of validation; RMSEP = root mean squared error of prediction.

**Table 3:** Quantitative performance of predicting LAC-MH content in tablet samples. The PLS was created for LAC-MH in the range of 0 to 100% *w*/*w*. ***Bold and italic text*** indicates the most effective fused model for quantifying LAC-MH overall.

Technique	Number of Factors	$R_{C}^{2}$	RMSEC/% *w*/*w*	$R_{V}^{2}$	RMSEV/% *w*/*w*	$R_{P}^{2}$	RMSEP/% *w*/*w*
A-532R-0	4	0.97	5.25	0.84	12.32	0.90	5.93
A-532R-1	4	0.98	17.67	0.68	17.67	0.74	9.39
A-532R-2	2	0.88	10.62	−0.12	33.03	−0.44	22.00
A-532B-0	2	0.48	22.62	0.01	31.12	−0.18	19.9
A-532B-1	1	0.12	29.34	−0.55	38.94	0.40	14.15
A-532B-2	1	0.31	25.91	0.08	29.97	−0.76	24.30
A-785R-0	3	0.91	9.54	0.17	28.40	0.89	6.13
A-785R-1	3	0.98	4.87	0.84	12.45	0.80	8.12
A-785R-2	2	0.93	8.08	0.06	30.18	0.57	12.06
***A-NIR***	***3***	***0.99***	***3.69***	***0.94***	***7.55***	***0.97***	***2.96***
B-785R-F	3	0.96	6.61	0.84	12.47	0.77	8.79
B-785R-S	3	0.96	6.11	0.86	11.86	0.70	10.11
B-NIR-F	2	0.93	8.11	0.64	18.77	0.92	5.26
B-NIR-S	2	0.98	4.91	0.91	9.27	0.92	5.16
A-532R-012	3	0.93	8.04	0.57	20.40	0.36	14.66
A-532R-0+NIR	2	0.94	7.40	0.81	13.57	0.82	7.85
A-532R-1+NIR	3	0.97	5.49	0.79	14.30	0.95	4.20
A-532R-2+NIR	3	0.99	2.11	0.92	8.63	0.95	4.21
A-532R-012+NIR	3	0.96	5.89	0.82	13.17	0.95	3.93
A-785R-012	3	0.97	5.44	0.78	14.50	0.78	8.54
A-785R-0+NIR	3	0.97	5.09	0.89	10.25	0.94	4.61
A-785R-1+NIR	3	0.98	4.62	0.90	9.67	0.94	4.40
A-785R-2+NIR	3	0.98	4.60	0.89	10.27	0.93	4.94
A-785R-012+NIR	3	0.97	5.48	0.88	11.00	0.96	3.72
B-785R-F+NIR	3	0.96	5.92	0.78	14.75	0.97	3.38
B-785R-S+NIR	3	0.96	5.84	0.84	12.44	0.91	5.39

RMSEC = root mean squared error of calibration; RMSEV = root mean squared error of validation; RMSEP = root mean squared error of prediction.

**Table 4:** Quantitative performance of predicting γ-IND content in tablet samples. The PLS was created for γ-IND in the range of 0 to 20.3% *w*/*w*. ***Bold and italic text*** indicates the most effective fused model for quantifying γ-IND overall.

Technique	Number of Factors	$R_{C}^{2}$	RMSEC/% *w*/*w*	$R_{V}^{2}$	RMSEV/% *w*/*w*	$R_{P}^{2}$	RMSEP/% *w*/*w*
A-532R-0	3	0.96	1.40	0.62	4.30	0.47	4.27
A-532R-1	2	0.93	1.80	0.48	5.07	0.85	2.27
A-532R-2	2	0.84	2.80	−0.02	7.07	0.33	4.81
A-532B-0	1	0.75	3.51	0.59	4.49	0.28	4.97
A-532B-1	3	0.94	1.74	0.78	3.29	0.40	4.55
A-532B-2	1	0.87	2.50	0.81	3.03	0.74	2.97
A-785R-0	2	0.96	1.46	0.89	2.28	0.97	1.07
A-785R-1	2	0.95	1.50	0.83	2.92	0.96	1.17
A-785R-2	2	0.90	2.24	0.67	4.04	0.95	1.37
A-NIR	5	0.99	0.69	0.94	1.67	0.96	1.10
B-785R-F	2	0.95	1.49	0.89	2.34	0.86	2.21
B-785R-S	2	0.97	1.28	0.90	2.24	0.91	1.79
B-NIR-F	3	0.69	3.90	0.27	6.00	0.52	4.07
B-NIR-S	4	0.97	1.18	0.76	3.45	0.91	1.78
A-532R-012	4	0.99	0.60	0.61	4.40	0.83	2.39
A-532R-0+NIR	4	0.98	1.05	0.64	4.23	0.66	3.44
A-532R-1+NIR	3	0.91	2.10	0.44	5.26	0.93	1.55
A-532R-2+NIR	3	0.96	1.32	0.60	4.46	0.55	3.93
A-532R-012+NIR	4	0.98	0.97	0.57	4.60	0.92	1.63
***A-785R-012***	***2***	***0.95***	***1.60***	***0.83***	***2.90***	***0.97***	***1.01***
A-785R-0+NIR	3	0.96	1.45	0.72	3.70	0.95	1.37
A-785R-1+NIR	3	0.96	1.38	0.72	3.71	0.92	1.71
A-785R-2+NIR	3	0.95	1.49	0.63	4.27	0.88	1.99
A-785R-012+NIR	3	0.95	0.91	0.84	2.78	0.95	1.32
B-785R-F+NIR	2	0.97	1.27	0.85	2.72	0.85	2.29
B-785R-S+NIR	2	0.98	1.19	0.87	2.56	0.92	1.65

RMSEC = root mean squared error of calibration; RMSEV = root mean squared error of validation; RMSEP = root mean squared error of prediction.

**Table 5:** Quantities of hydroxypropyl methylcellulose (HPMC), anatase titanium dioxide (a-TD), lactose monohydrate (LAC-MH), and gamma indomethacin (γ-IND) within pseudo-bilayer (BL) tablet samples.

Dataset Type	HPMC/*w*/*w* %	a-TD/*w*/*w* %	LAC-MH/*w*/*w* %	γ-IND/*w*/*w* %	Thickness/mm	Tablet Sample ID
Model set	12.54	0	87.46	0	3.45	1
	28.03	0	71.97	0	4.3	3
	42.36	0	57.64	0	5.55	5
	17.72	1.98	80.30	0	3.8	6
	32.35	3.61	64.04	0	4.9	8
	37.52	4.19	58.29	0	5.5	9
	12.82	0	69.46	17.72	3.45	10
	28.55	0	56.93	14.52	4.3	12
	42.98	0	45.45	11.57	5.55	14
	18.09	2.02	63.65	16.24	3.8	15
	32.90	3.65	50.55	12.90	4.9	17
	38.08	4.25	45.95	11.72	5.5	18
Test set	19.38	0	80.62	0	3.75	2
	35.74	0	64.26	0	4.9	4
	25.26	2.82	71.92	0	4.25	7
	19.69	0	64.00	16.31	3.75	11
	36.33	0	50.73	12.94	4.9	13
	25.84	2.88	56.78	14.50	4.25	16

**Table 6:** Quantities of hydroxypropyl methylcellulose (HPMC), anatase titanium dioxide (a-TD), lactose monohydrate, and gamma indomethacin (γ-IND) mixtures within tablet samples.

Dataset Type	HPMC/*w*/*w* %	a-TD/*w*/*w* %	LAC-MH/*w*/*w* %	γ-IND/*w*/*w* %	Tablet Sample ID
Model set	51.20	0	48.80	0	1
	44.78	5.00	40.02	10.20	4
	100	0	0	0	5
	89.80	10.20	0	0	6
	0	0	100	0	7
	0	0	79.69	20.31	8
	25.02	0	74.98	0	9
	22.33	2.49	59.91	15.27	12
	74.97	0	25.03	0	13
	65.90	7.35	26.75	0	14
	74.50	0	20.32	5.18	15
Test set	44.95	5.00	50.05	0	2
	50.34	0	39.58	10.08	3
	22.58	2.52	74.90	0	10
	25.59	0	59.30	15.11	11
	66.85	7.45	20.48	5.22	16

## Data Availability

Data is contained within the article or Supplementary Materials.

## Supplementary Materials

The following supporting information can be downloaded at: https://www.mdpi.com/article/10.3390/ph17121659/s1, Figure S1: Illustration of Raman setup 1 coupled to Probe A; Figure S2: Illustration of Raman setup 2 coupled to Probe B; Figure S3: Illustration of Raman setup 3 coupled to Probe A; Figure S4: Raw spectra of tablet formulations collected using the following probe configurations: (a) A-532R-0, (b) A-532R-1, (c) A-532R-2, (d) A-785R-0, (e) A-785R-1, (f) A-785R-2, (g) B-785R-F, (h) B-785R-S, (i) A-NIR, (j) B-NIR-F, and (k) B-NIR-S; Figure S5: Scores plots of the first principal component and second principal components of all measurement configurations (A-532R-0, A-532R-1, A-532R-2, A-785R-0, A-785R-1, A-785R-2, A-NIR, B-785R-F, B-785R-S, B-NIR-F, B-NIR-S); Figure S6: Reference raw A-532R-0 spectra of HPMC (red), a-TD (blue), LAC-MH (gold), and γ-IND (purple) compared against first (PC1) and second (PC2) principal components loadings plot of tablet formulations; Figure S7: Reference raw A-532R-1 spectra of HPMC (red), a-TD (blue), LAC-MH (gold), and γ-IND (purple) compared against first (PC1) and second (PC2) principal components loadings plot of tablet formulations; Figure S8: Reference raw A-532R-2 spectra of HPMC (red), a-TD (blue), LAC-MH (gold), and γ-IND (purple) compared against first (PC1) and second (PC2) principal components loadings plot of tablet formulations; Figure S9: Reference raw A-785R-0 spectra of HPMC (red), a-TD (blue), LAC-MH (gold), and γ-IND (purple) compared against first (PC1) and second (PC2) principal components loadings plot of tablet formulations; Figure S10: Reference raw A-785R-1 spectra of HPMC (red), a-TD (blue), LAC-MH (gold), and γ-IND (purple) compared against first (PC1) and second (PC2) principal components loadings plot of tablet formulations; Figure S11: Reference raw A-785R-2 spectra of HPMC (red), a-TD (blue), LAC-MH (gold), and γ-IND (purple) compared against first (PC1) and second (PC2) principal components loadings plot of tablet formulations; Figure S12: Reference preprocessed A-NIR spectra of HPMC (red), a-TD (blue), LAC-MH (gold), and γ-IND (purple) compared against first (PC1) and second (PC2) principal components loadings plot of tablet formulations; Figure S13: Reference preprocessed B-785R-F spectra of HPMC (red), a-TD (blue), LAC-MH (gold), and γ-IND (purple) compared against first (PC1) and second (PC2) principal components loadings plot of tablet formulations; Figure S14: Reference raw B-785R-F spectra of HPMC (red), a-TD (blue), LAC-MH (gold), and γ-IND (purple) compared against first (PC1) and second (PC2) principal components loadings plot of tablet formulations; Figure S15: Reference preprocessed B-NIR-F spectra of HPMC (red), a-TD (blue), LAC-MH (gold), and γ-IND (purple) compared against first (PC1) and second (PC2) principal components loadings plot of tablet formulations; Figure S16: Reference preprocessed B-NIR-S spectra of HPMC (red), a-TD (blue), LAC-MH (gold), and γ-IND (purple) compared against first (PC1) and second (PC2) principal components loadings plot of tablet formulations; Figure S17: Reference preprocessed A-NIR spectra of KBr (teal), HPMC (red), a-TD (blue), LAC-MH (gold), and γ-IND (purple) compared against regression coefficients (3 factors) of A-NIR PLS model predicting for HPMC content in tablet samples; Figure S18: Reference preprocessed A-532R-0 spectra of KBr (teal), HPMC (red), a-TD (blue), LAC-MH (gold), and γ-IND (purple) compared against regression coefficients (1 factor) of A-532R-0 PLS model predicting for a-TD content in tablet samples; Figure S19: Reference preprocessed B-785R-F spectra of HPMC (red), a-TD (blue), LAC-MH (gold), and γ-IND (purple) compared against regression coefficients (1 factor) of A-B785R-F PLS model predicting for a-TD content in tablet samples; Figure S20: Reference preprocessed A-NIR spectra of KBr (teal), HPMC (red), a-TD (blue), LAC-MH (gold), and γ-IND (purple) compared against regression coefficients (3 factors) of A-NIR PLS model predicting for LAC-MH content in tablet samples; Figure S21: Reference preprocessed A-785R-0 spectra of KBr (teal), HPMC (red), a-TD (blue), LAC-MH (gold), and γ-IND (purple) compared against regression coefficients (2 factors) of A-785R-012 PLS model predicting for γ-IND content in tablet samples; Figure S22: Reference preprocessed A-785R-0 spectra of KBr (teal), HPMC (red), a-TD (blue), LAC-MH (gold), and γ-IND (purple) compared against regression coefficients (2 factors) of A-785R-0 PLS model for predicting HPMC content in bilayers; Figure S23: Reference preprocessed A-785R-0 spectra of KBr (teal), HPMC (red), a-TD (blue), LAC-MH (gold), and γ-IND (purple) compared against regression coefficients (2 factors) of A-785R-0 PLS model for predicting a-TD content in bilayers; Figure S24: Reference preprocessed A-532R-0 spectra of KBr (teal), HPMC (red), a-TD (blue), LAC-MH (gold), and γ-IND (purple) compared against regression coefficients (2 factors) of A-532R-012 PLS model for predicting LAC-MH content in bilayers; Figure S25: Reference preprocessed A-532R-0 spectra of KBr (teal), HPMC (red), a-TD (blue), LAC-MH (gold), and γ-IND (purple) compared against regression coefficients (2 factors) of A-532R-012 PLS model for predicting γ-IND content in bilayers; Figure S26: Reference preprocessed A-785R-0 spectra of KBr (teal), HPMC (red), a-TD (blue), LAC-MH (gold), and γ-IND (purple) compared against regression coefficients (2 factors) of A-785R-2 PLS model for predicting bilayer thickness; Figure S27: Plot of actual vs. prediction HPMC content in tablet samples using PLS model developed with A-NIR data with 3 factors; Figure S28: Plot of actual vs. prediction a-TD content in tablet samples using PLS model developed with B-785R+NIR data with 1 factor; Figure S29: Plot of actual vs. prediction LAC-MH content in tablet samples using PLS model developed with A-NIR data with 3 factors; Figure S30: Plot of actual vs. prediction γ-IND content in tablet samples using PLS model developed with A- data with 2 factors; Figure S31: Plot of actual vs. prediction HPMC content in BL samples using PLS model developed with A-785R-0 data with 5 factors; Figure S32: Plot of actual vs. prediction a-TD content in BL samples using PLS model developed with A-785R-0 data with 1 factor; Figure S33: Plot of actual vs. prediction LAC-MH content in BL samples using PLS model developed with A-532R-0 data with 4 factors; Figure S34: Plot of actual vs. prediction γ-IND content in BL samples using PLS model developed with A-532B-2 data with 2 factors; Figure S35: Plot of actual vs. prediction thickness of BL samples using PLS model developed with A-785R-2 data with 3 factors; Table S1: Table of name and descriptions of different configurations of the fibre optic probes; Table S2: Quantitative performance of predicting HPMC content in bilayer tablet samples. The PLS model was created for 12.8 to 43% *w*/*w*. Techniques with bold characters were the best valid performing technique within each wavelength. Techniques with italic and bold characters were the best performing technique overall; Table S3: Quantitative performance of predicting a-TD content in bilayer tablet samples. The PLS model was created for 0 to 4.25% *w*/*w*. Techniques with bold characters were the best valid performing technique within each wavelength. Techniques with italic and bold characters were the best performing technique overall; Table S4: Quantitative performance of predicting LAC-MH content in bilayer tablet samples. The PLS model was created for 45.45 to 87.46% *w*/*w*. Techniques with bold characters were the best valid performing technique within each wavelength. Techniques with italic and bold characters were the best performing technique overall; Table S5: Quantitative performance of predicting γ-IND content in bilayer tablet samples. The PLS model was created for 0to 17.72% *w*/*w*. Techniques with bold characters were the best valid performing technique within each wavelength. Techniques with italic and bold characters were the best performing technique overall; Table S6: Quantitative performance of predicting thickness of bilayer tablet samples. The PLS model was created for 3.45 to 5.55 mm. Techniques with bold characters were the best valid performing technique within each wavelength. Techniques with italic and bold characters were the best performing technique overall.
