# Supplementary material for: A Quantitative Chemometric Study of Pharmaceutical Tablet Formulations Using Multi-Spectroscopic Fibre Optic Probes
Source: Pharmaceuticals (Basel). 2024 Dec 9;17(12):1659. doi: 10.3390/ph17121659 (PMC11677398; doi:10.3390/ph17121659)
Supplement: Supplementary file 1 [file pharmaceuticals-17-01659-s001.zip › pharmaceuticals-3327285-supplementary.pdf]

A Quantitative Chemometric Study of Pharmaceutical Mixtures Using Multi-Spectroscopic Fibre Optic Probes

Peter III J. G. Remoto[1], Sara J. Fraser-Miller[1*], Keith C. Gordon[1*]

[1] The Dodd-Walls Centre for Photonic and Quantum Technologies, Department of Chemistry, University of Otago, Dunedin, 9016, New Zealand

* Correspondence authors: sara.miller@otago.ac.nz; keith.gordon@otago.ac.nz

Supporting Information

Table S1. Table of name and descriptions of different configurations of the fibre optic probes.

Configuration name	Probe	Spectroscopic technique	Light source / nm	Description
A-532R-0	A	RS	532	532 nm light source fibre line and light collector fibre line bundled within the same probe tip (i.e., approximately no spatial offset).
A-532R-1	A	SORS	532	532 nm light source fibre line is ~2.5 mm away from the light collector fibre line.
A-532R-2	A	SORS	532	532 nm light source (output) line is ~5 mm away from the light collector (input) fibre line.
A-785R-0	A	RS	785	785 nm light source fibre line and light collector fibre line bundled within the same probe tip (i.e., approximately no spatial offset).
A-785R-1	A	SORS	785	785 nm light source (output) line is ~2.5 mm away from the light collector (input) fibre line.
A-785R-2	A	SORS	785	785 nm light source (output) line is ~5.0 mm away from the light collector (input) fibre line.
A-NIR	A	NIRS	900 – 1745	All three probe tips contain NIR light source fibre lines and light collector fibre line for reflectance NIR spectroscopic measurements.

B-785R-F	B	RS	785	Unmodified Probe tip containing 785 light source and collector.
B-785R-S	B	RS	785	Similar to B-785R-F but the probe tip was equipped with an accessory which redirects light to be outputted and collected perpendicular to the probe tip (i.e., side-on configuration).
B-NIR-F	B	NIRS	900 – 1745	Unmodified Probe tip containing NIR light source and collector for reflectance NIR spectroscopy
B-NIR-S	B	NIRS	900 – 1745	Similar to B-NIR-F but the probe tip was equipped with an accessory which redirects light to be outputted and collected perpendicular to the probe tip (i.e., side-on configuration).

* NIRS = Near-Infrared Spectroscopy; RS = Raman Spectroscopy; SORS = Spatially Offset Raman Spectroscopy

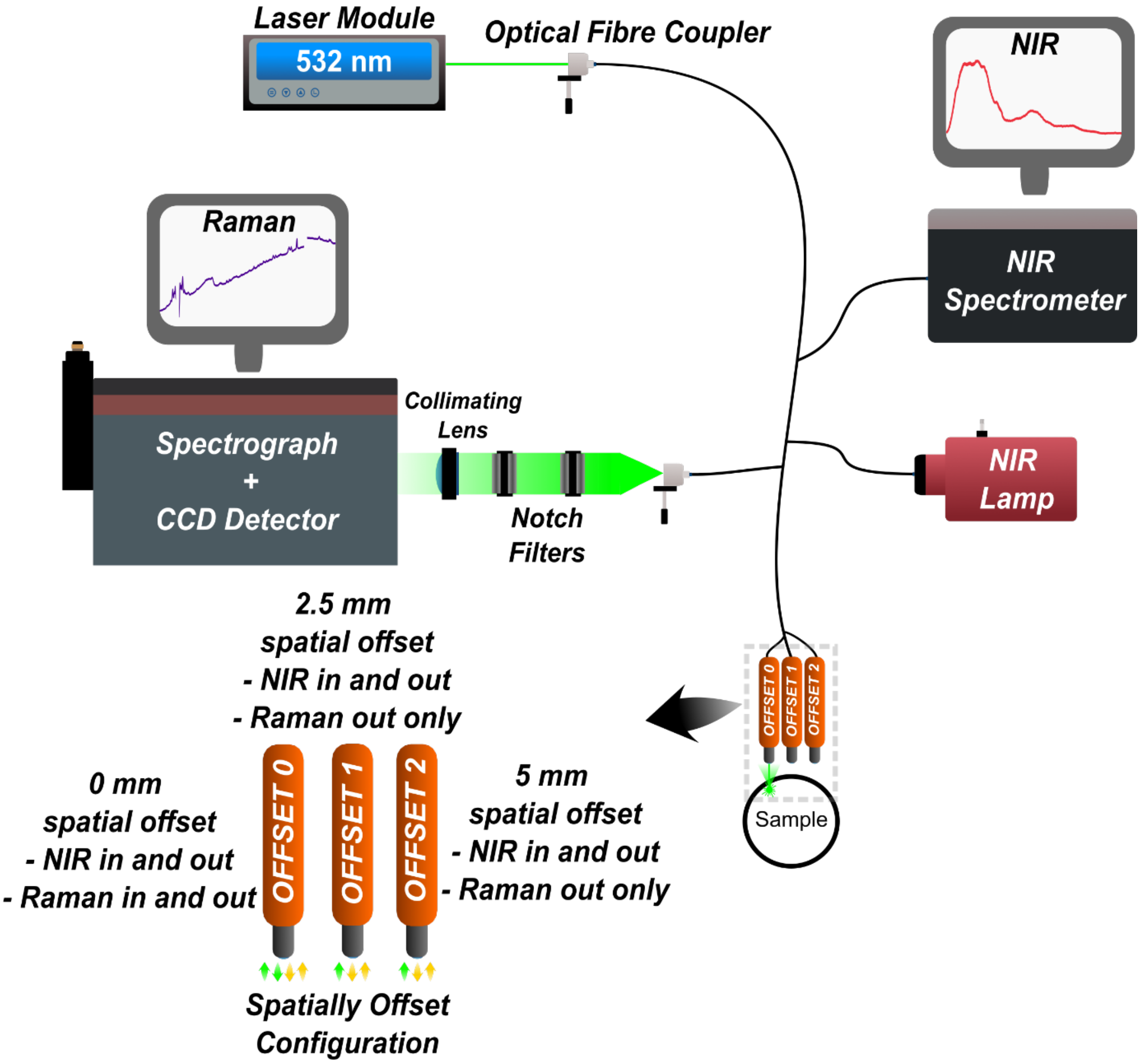

Figure S1. Illustration of Raman setup 1 coupled to Probe A.

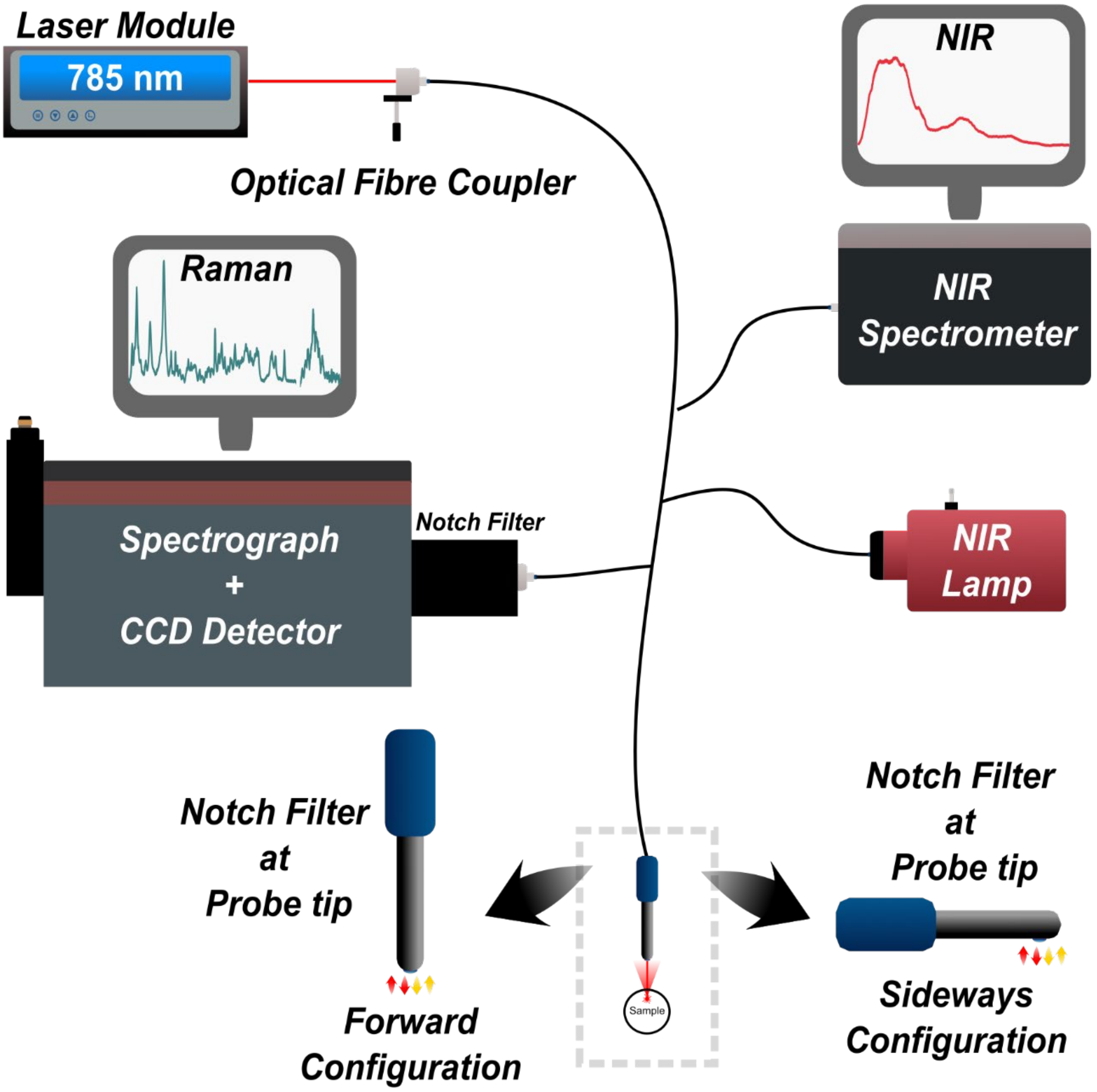

Figure S2. Illustration of Raman setup 2 coupled to Probe B.

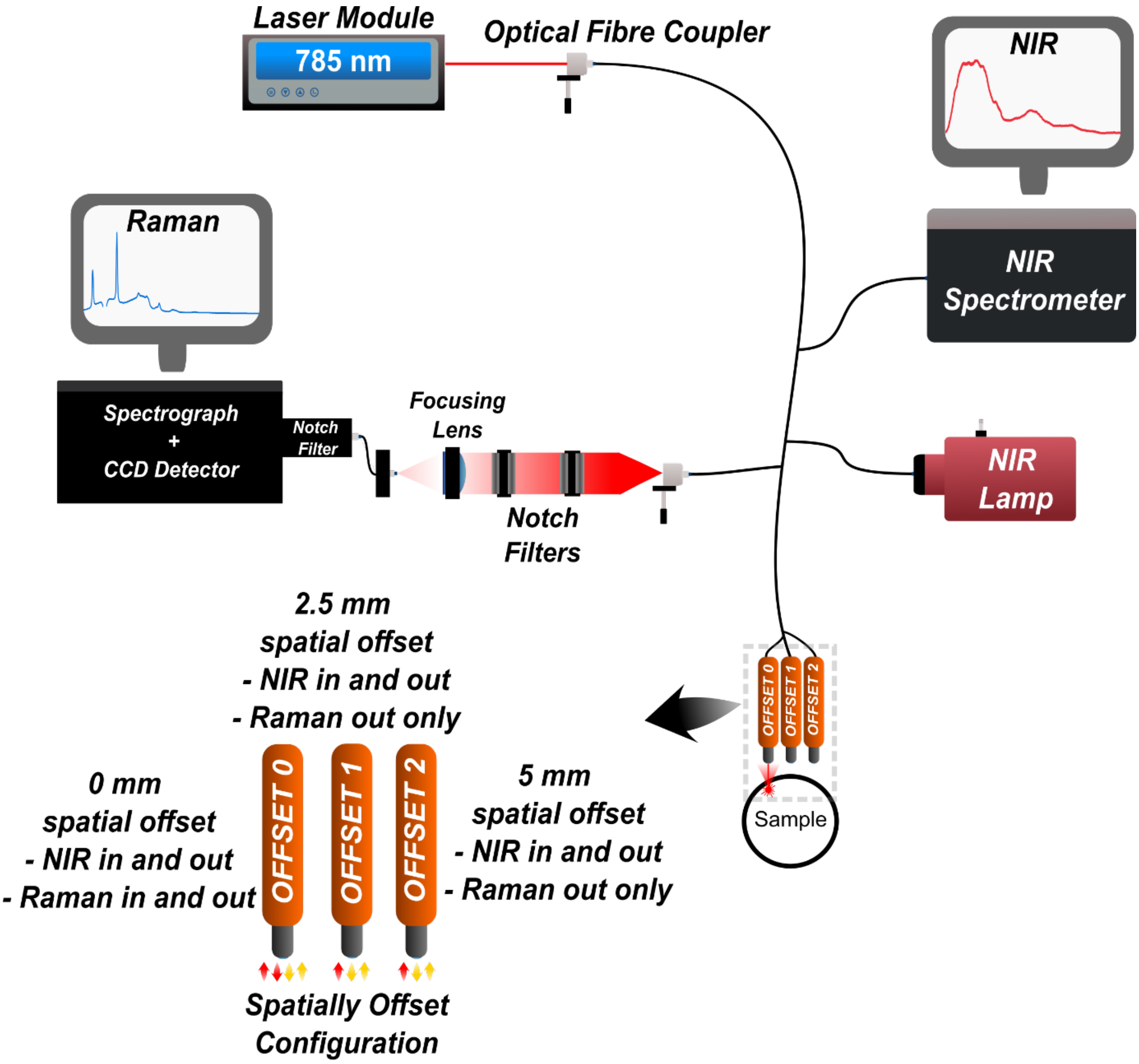

Figure S3. Illustration of Raman setup 3 coupled to Probe A.

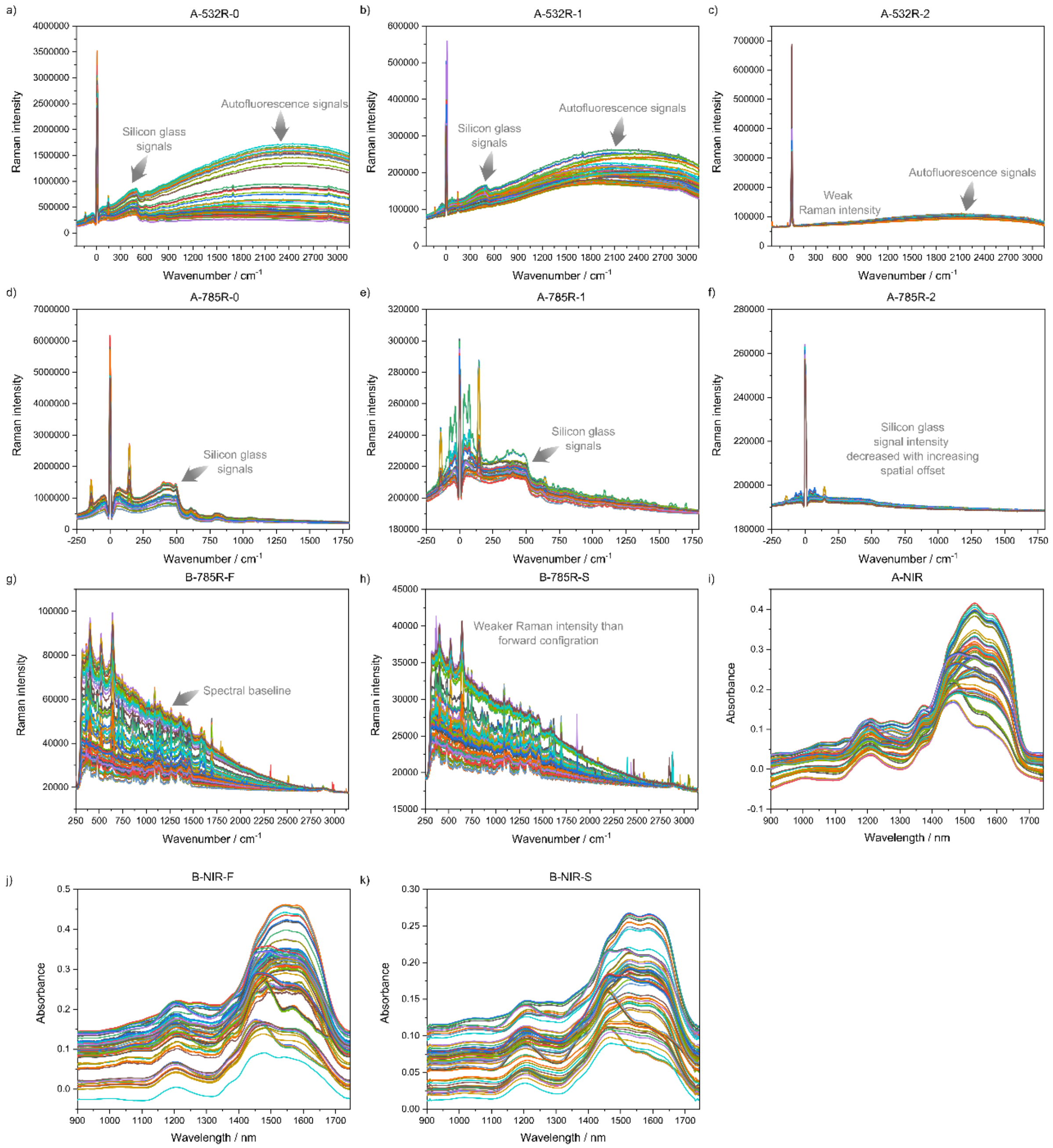

Figure S4. Raw spectra of tablet formulations collected using the following probe configurations: a) A-532R-0, b) A-532R-1, c) A-532R-2, d) A-785R-0, e) A-785R-1, f) A-785R-2, g) B-785R-F, h) B-785R-S, i) A-NIR, j) B-NIR-F, and k) B-NIR-S.

Potassium bromide (KBr) exhibits very weak Raman scattering due to its relatively simple structure (K-Br) and localised vibration, which leads to low change of polarisability during vibration. This can be taken advantage of to be used to observe persistent artefacts and detect signals which arise from the make of the tool, such as silicon glass.

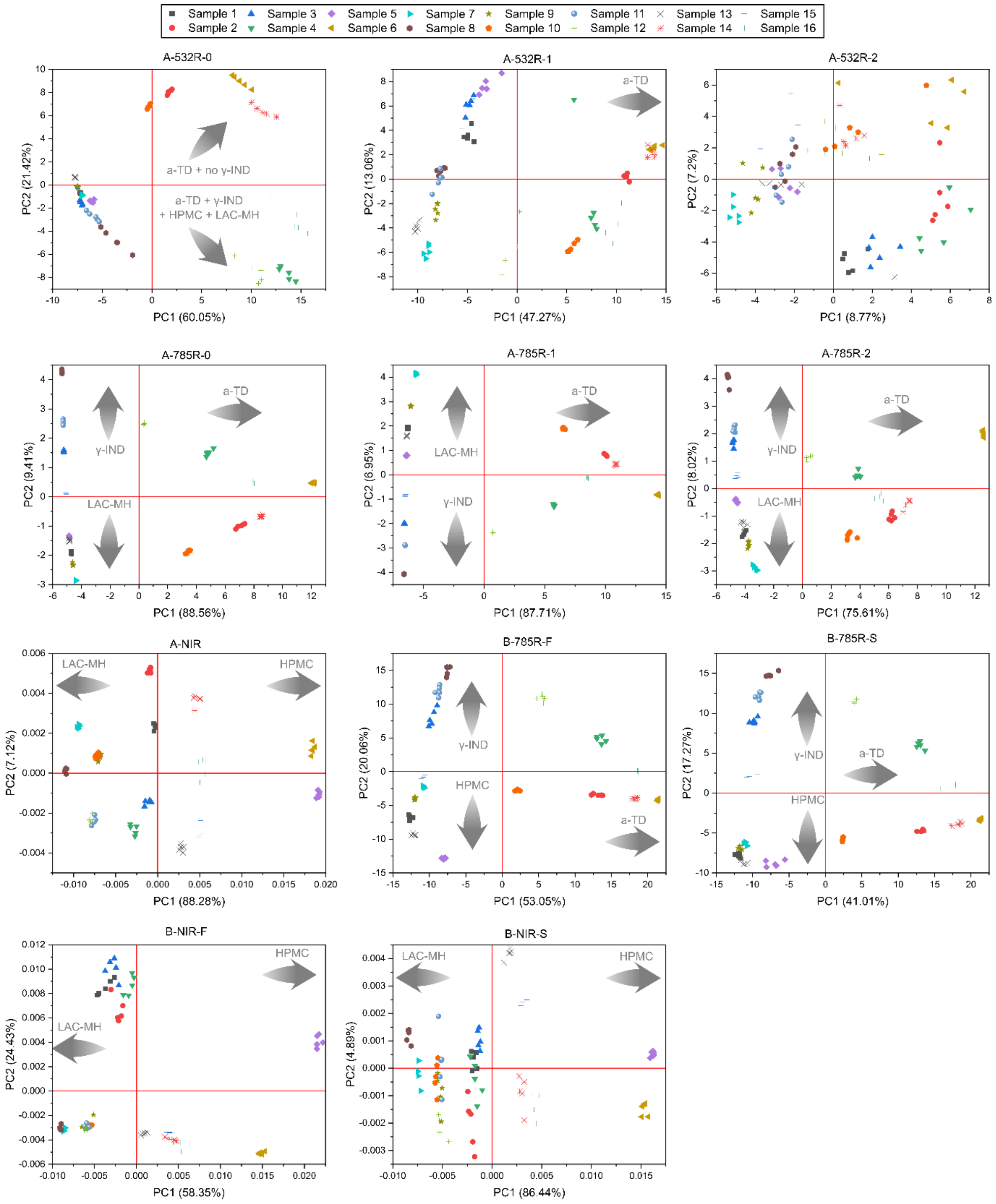

Figure S5. Scores plots of the first principal component and second principal components of all measurement configurations (A-532R-0, A-532R-1, A-532R-2, A-785R-0, A-785R-1, A-785R-2, A-NIR, B-785R-F, B-785R-S, B-NIR-F, B-NIR-S).

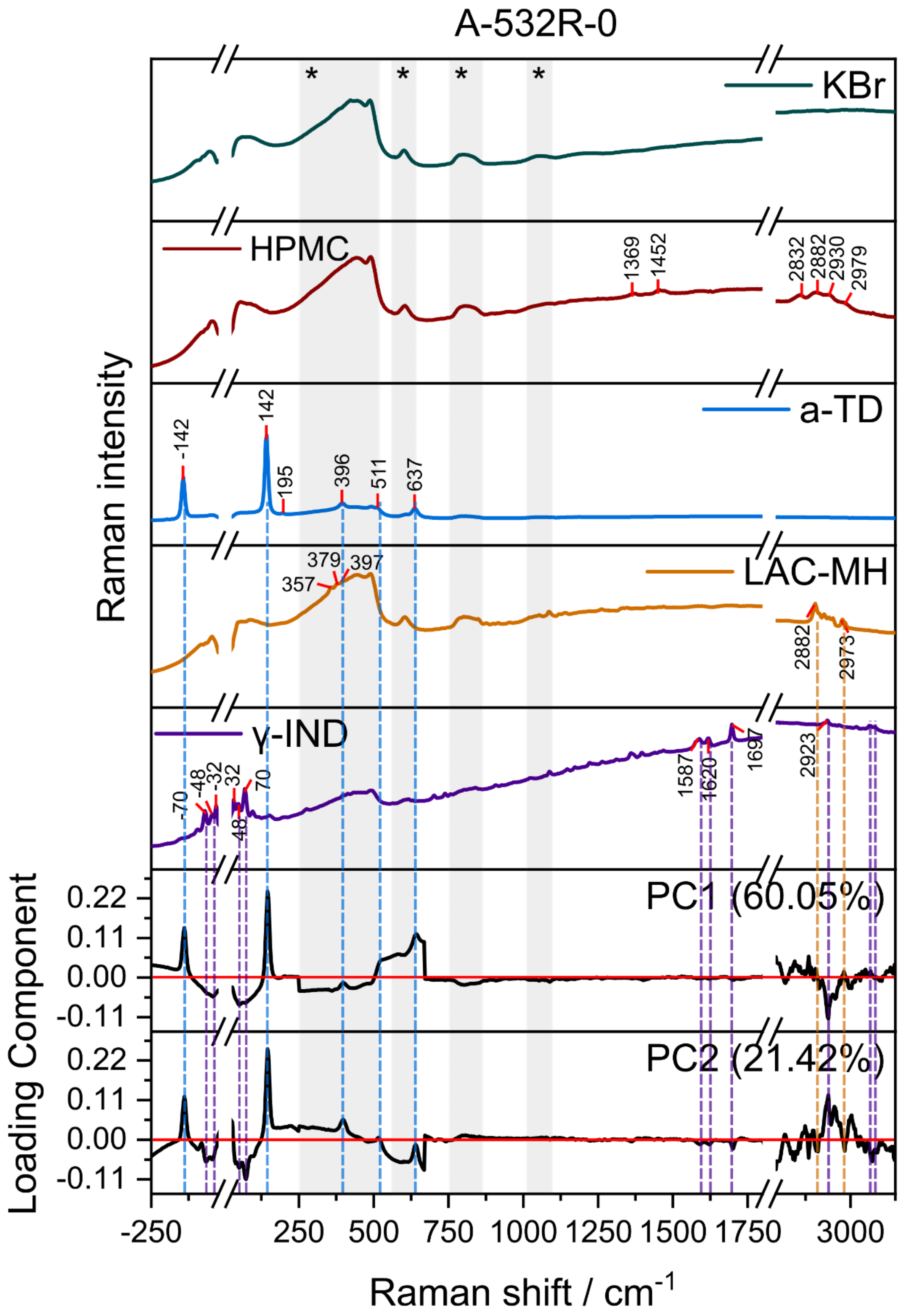

Figure S6. Reference raw A-532R-0 spectra of HPMC (red), a-TD (blue), LAC-MH (gold), and γ-IND (purple) compared against first (PC1) and second (PC2) principal components loadings plot of tablet formulations.

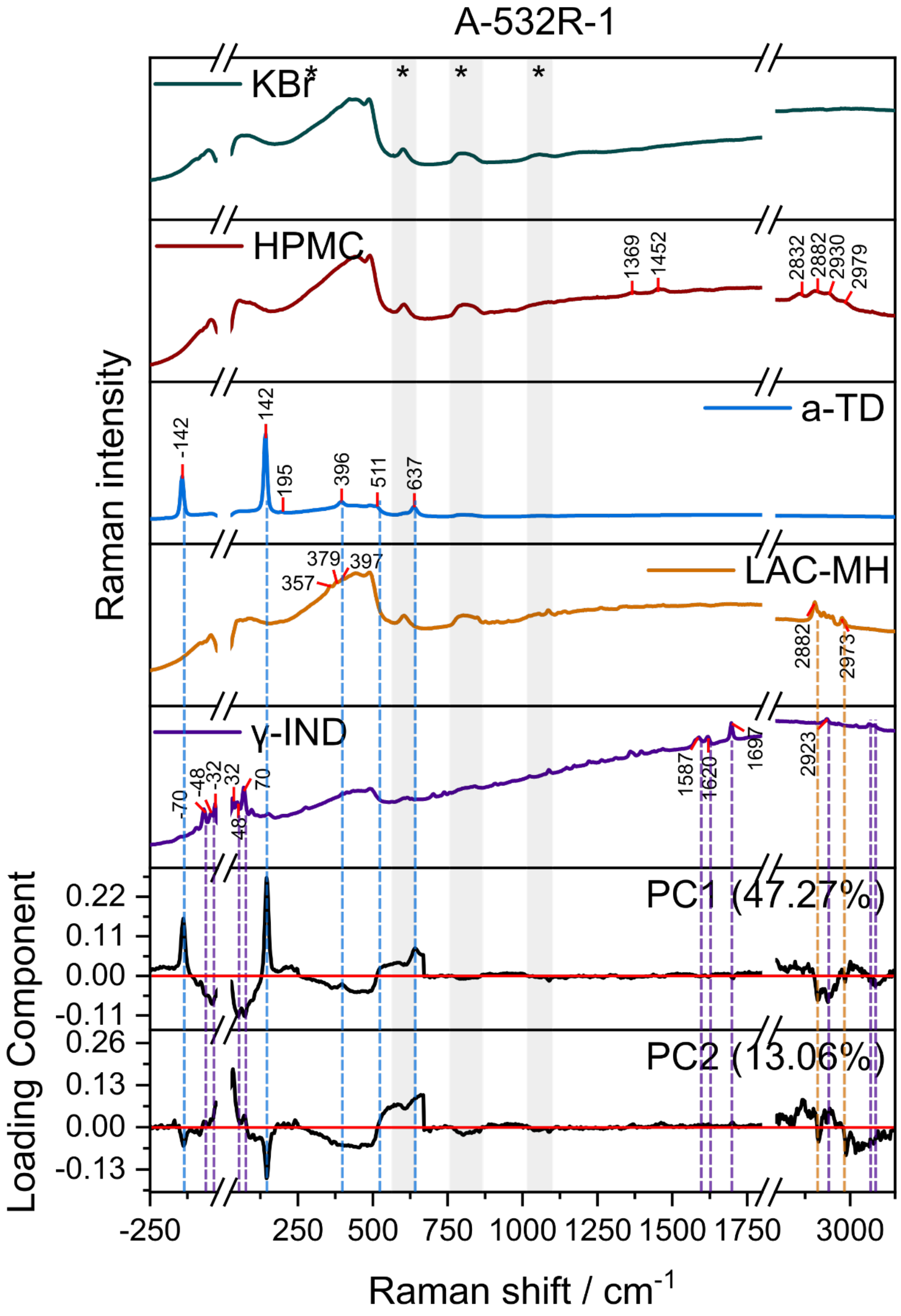

Figure S7. Reference raw A-532R-1 spectra of HPMC (red), a-TD (blue), LAC-MH (gold), and γ-IND (purple) compared against first (PC1) and second (PC2) principal components loadings plot of tablet formulations.

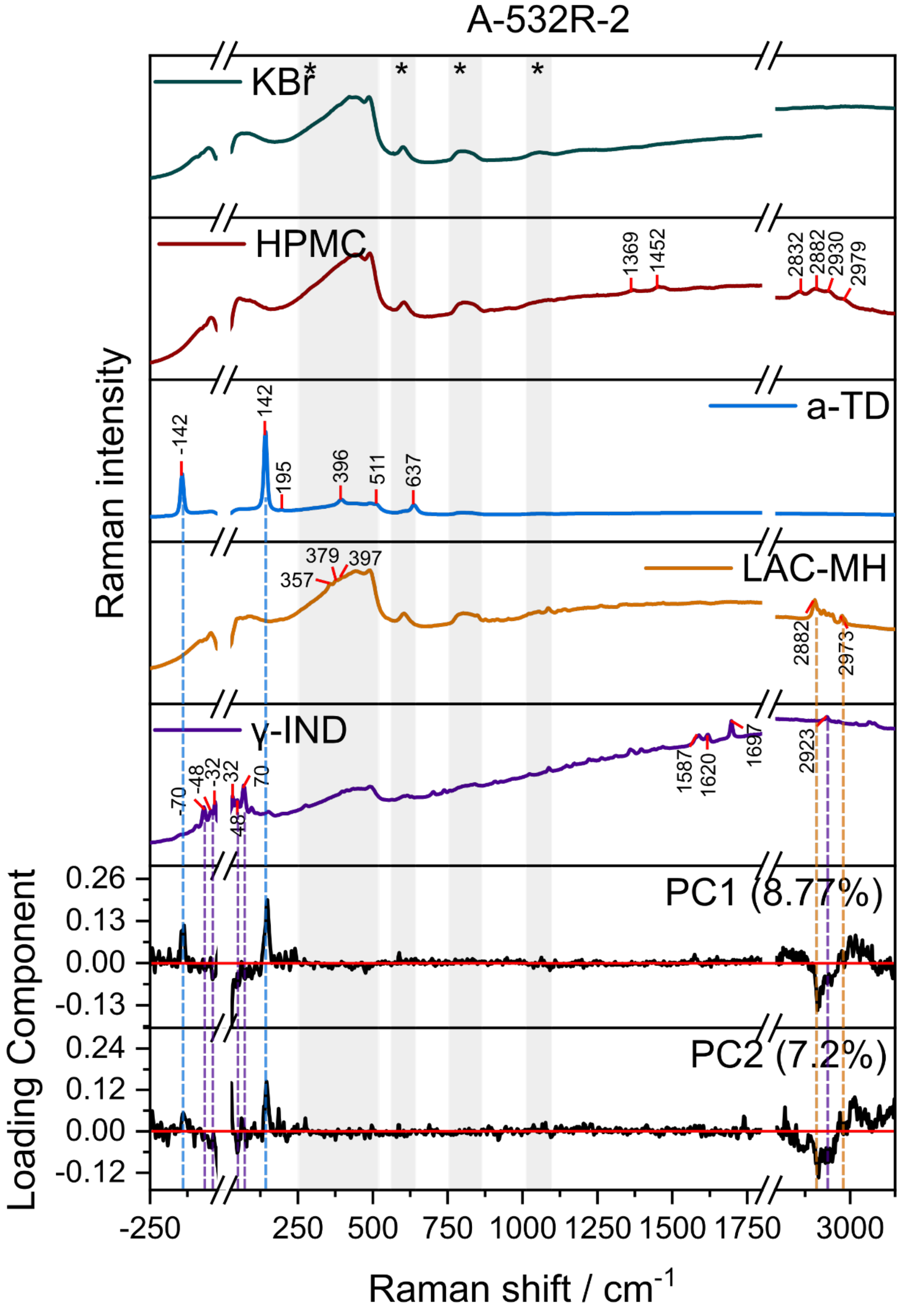

Figure S8. Reference raw A-532R-2 spectra of HPMC (red), a-TD (blue), LAC-MH (gold), and γ-IND (purple) compared against first (PC1) and second (PC2) principal components loadings plot of tablet formulations.

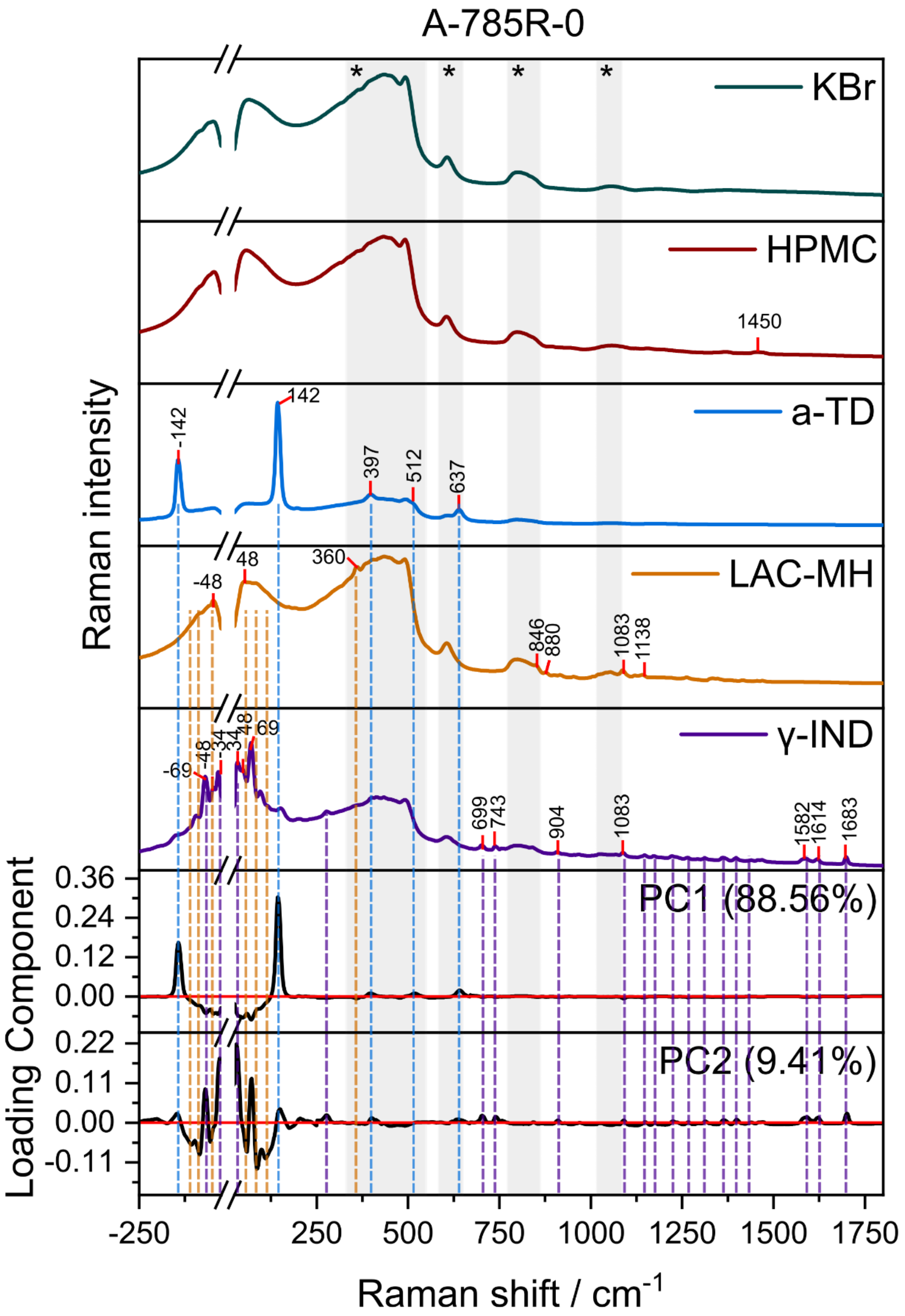

Figure S9. Reference raw A-785R-0 spectra of HPMC (red), a-TD (blue), LAC-MH (gold), and γ-IND (purple) compared against first (PC1) and second (PC2) principal components loadings plot of tablet formulations.

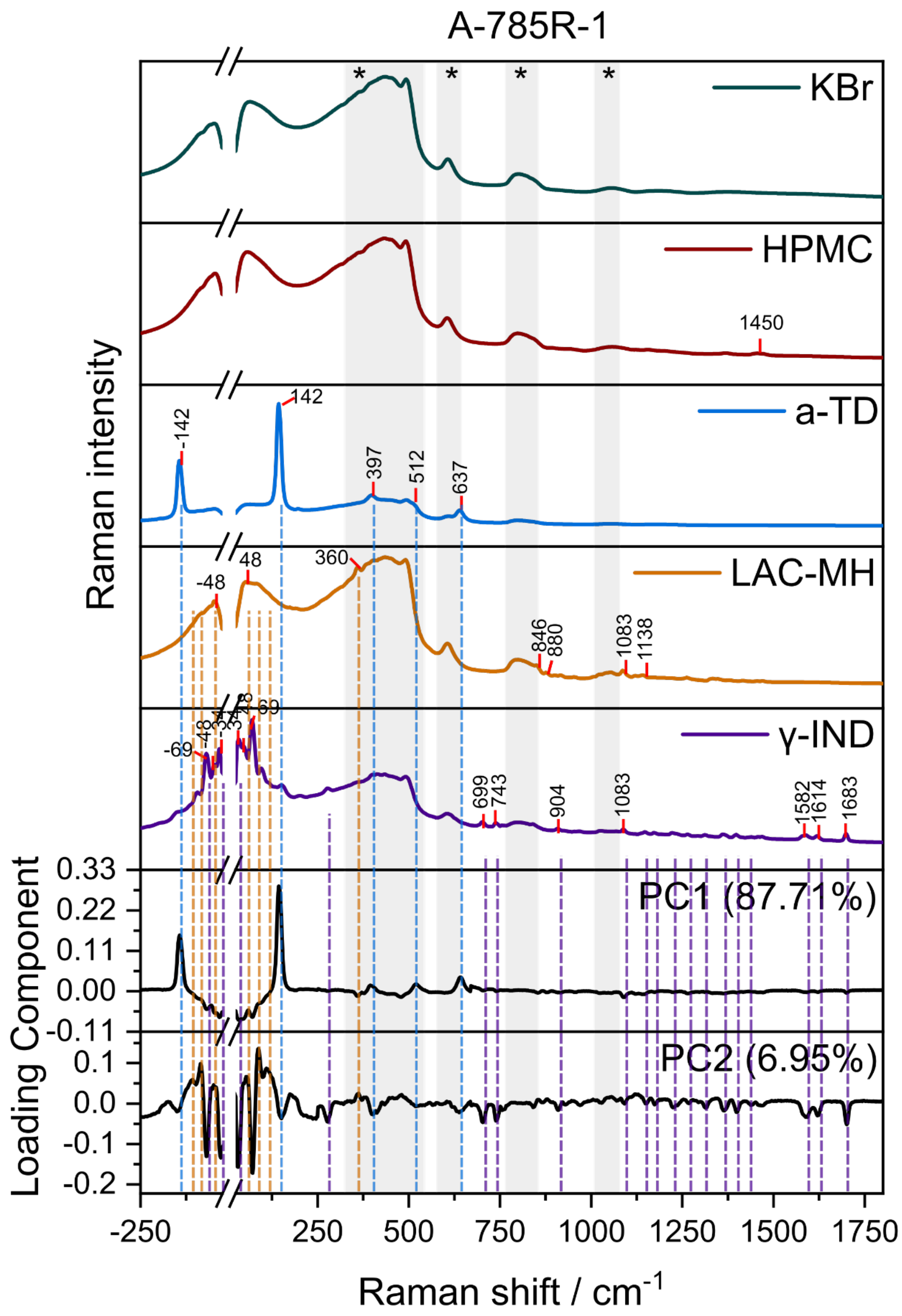

Figure S10. Reference raw A-785R-1 spectra of HPMC (red), a-TD (blue), LAC-MH (gold), and γ-IND (purple) compared against first (PC1) and second (PC2) principal components loadings plot of tablet formulations.

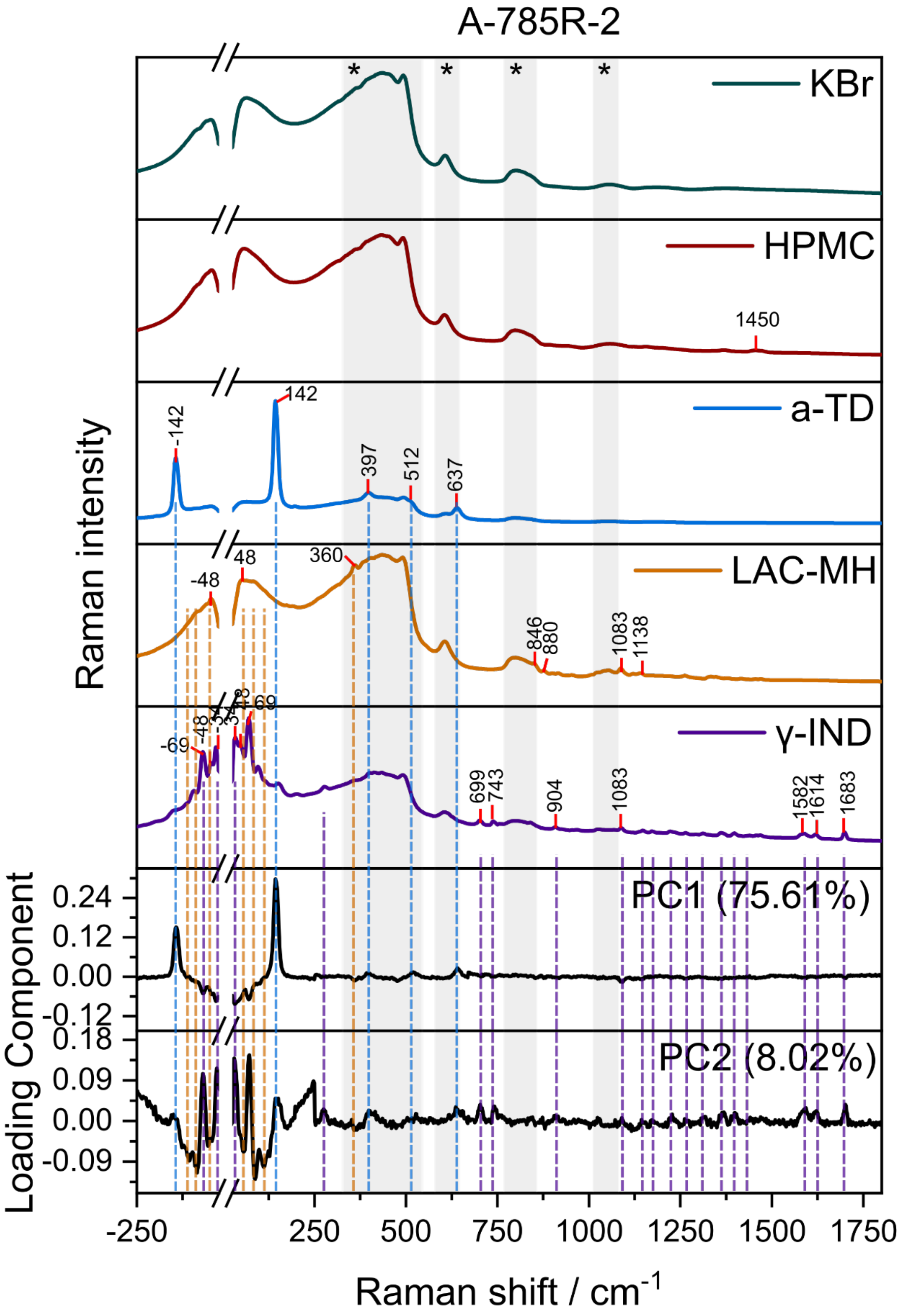

Figure S11. Reference raw A-785R-2 spectra of HPMC (red), a-TD (blue), LAC-MH (gold), and γ-IND (purple) compared against first (PC1) and second (PC2) principal components loadings plot of tablet formulations.

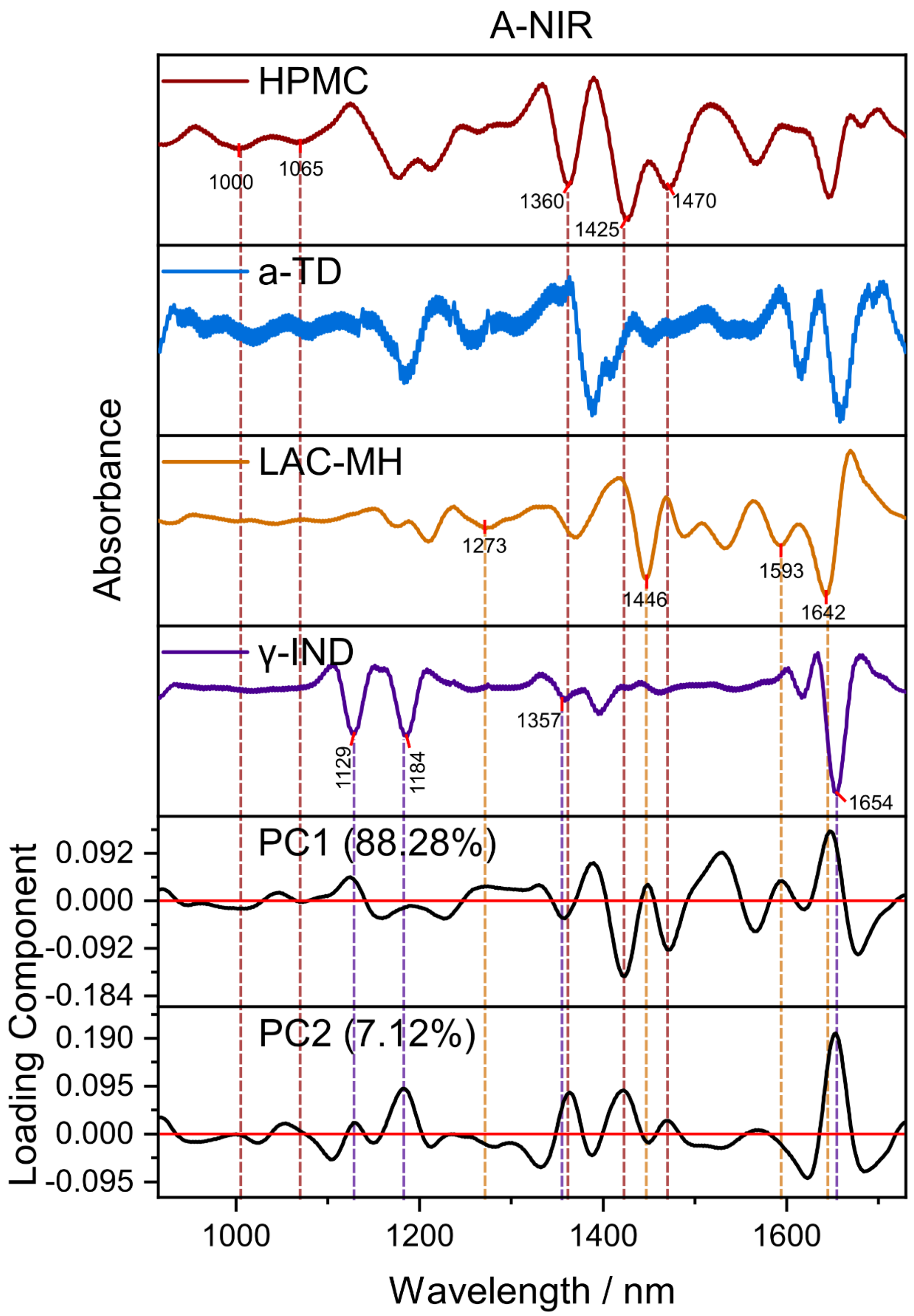

Figure S12. Reference preprocessed A-NIR spectra of HPMC (red), a-TD (blue), LAC-MH (gold), and γ-IND (purple) compared against first (PC1) and second (PC2) principal components loadings plot of tablet formulations.

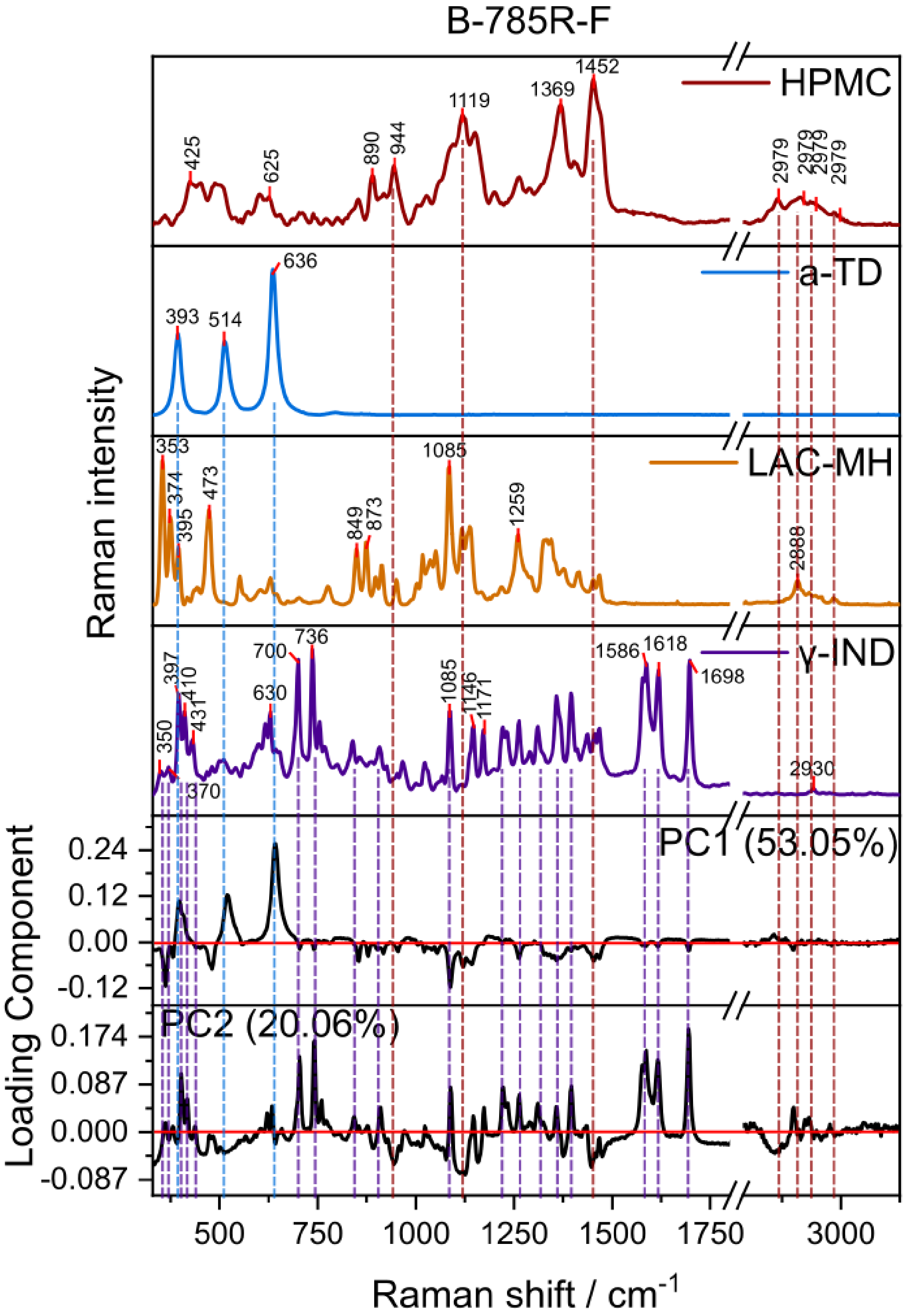

Figure S13. Reference preprocessed B-785R-F spectra of HPMC (red), a-TD (blue), LAC-MH (gold), and γ-IND (purple) compared against first (PC1) and second (PC2) principal components loadings plot of tablet formulations.

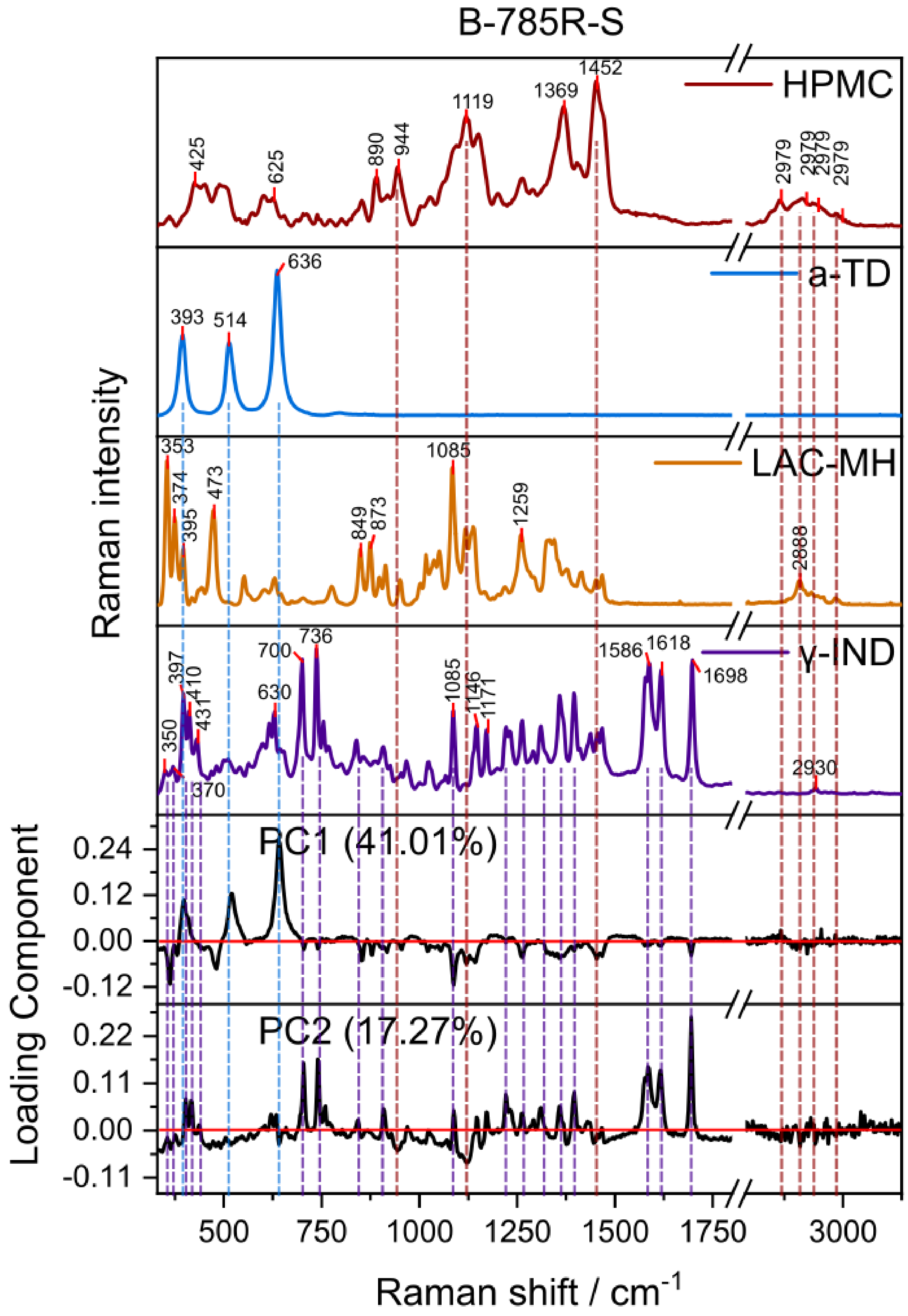

Figure S14. Reference raw B-785R-F spectra of HPMC (red), a-TD (blue), LAC-MH (gold), and γ-IND (purple) compared against first (PC1) and second (PC2) principal components loadings plot of tablet formulations.

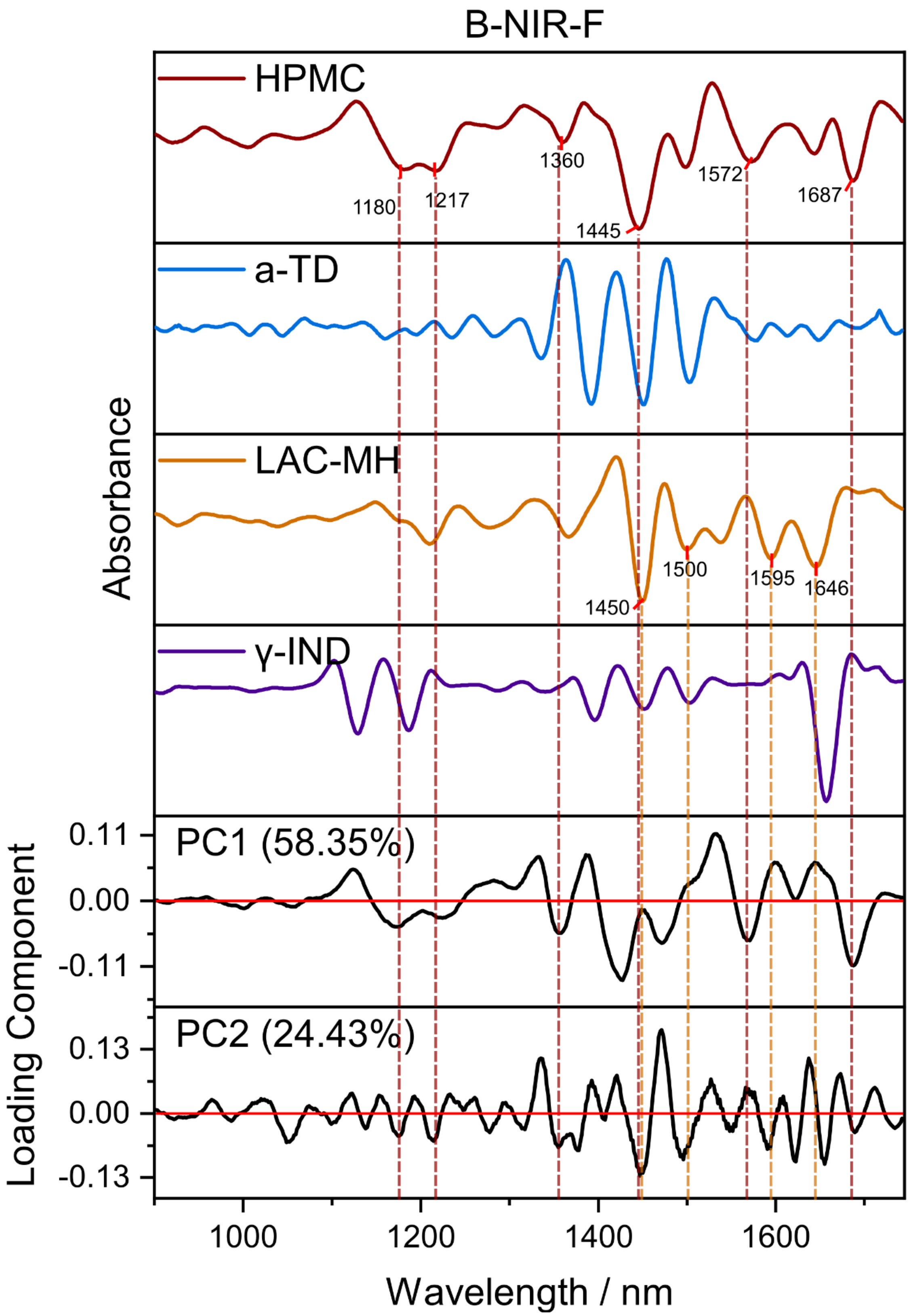

Figure S15. Reference preprocessed B-NIR-F spectra of HPMC (red), a-TD (blue), LAC-MH (gold), and γ-IND (purple) compared against first (PC1) and second (PC2) principal components loadings plot of tablet formulations.

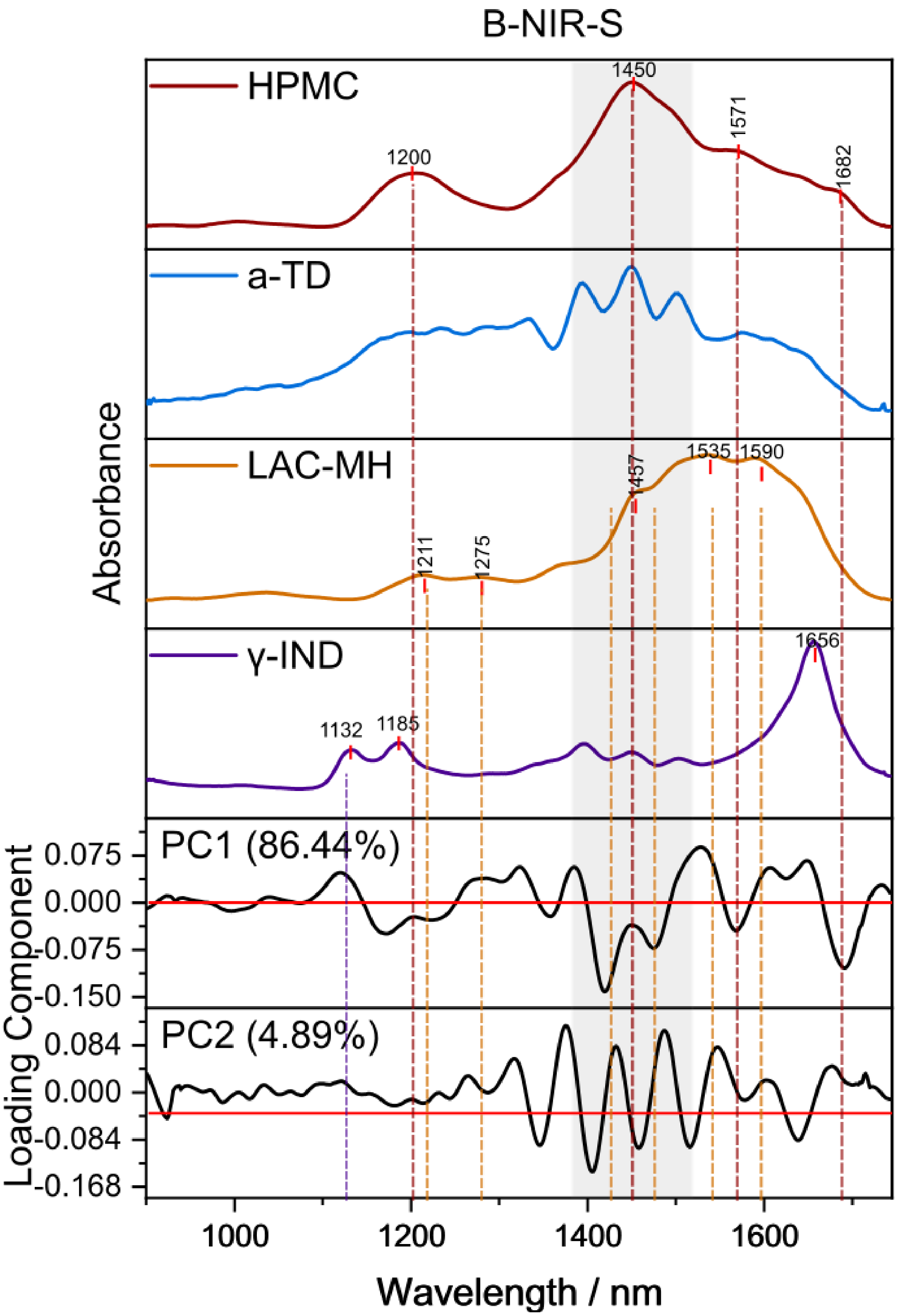

Figure S16. Reference preprocessed B-NIR-S spectra of HPMC (red), a-TD (blue), LAC-MH (gold), and γ-IND (purple) compared against first (PC1) and second (PC2) principal components loadings plot of tablet formulations.

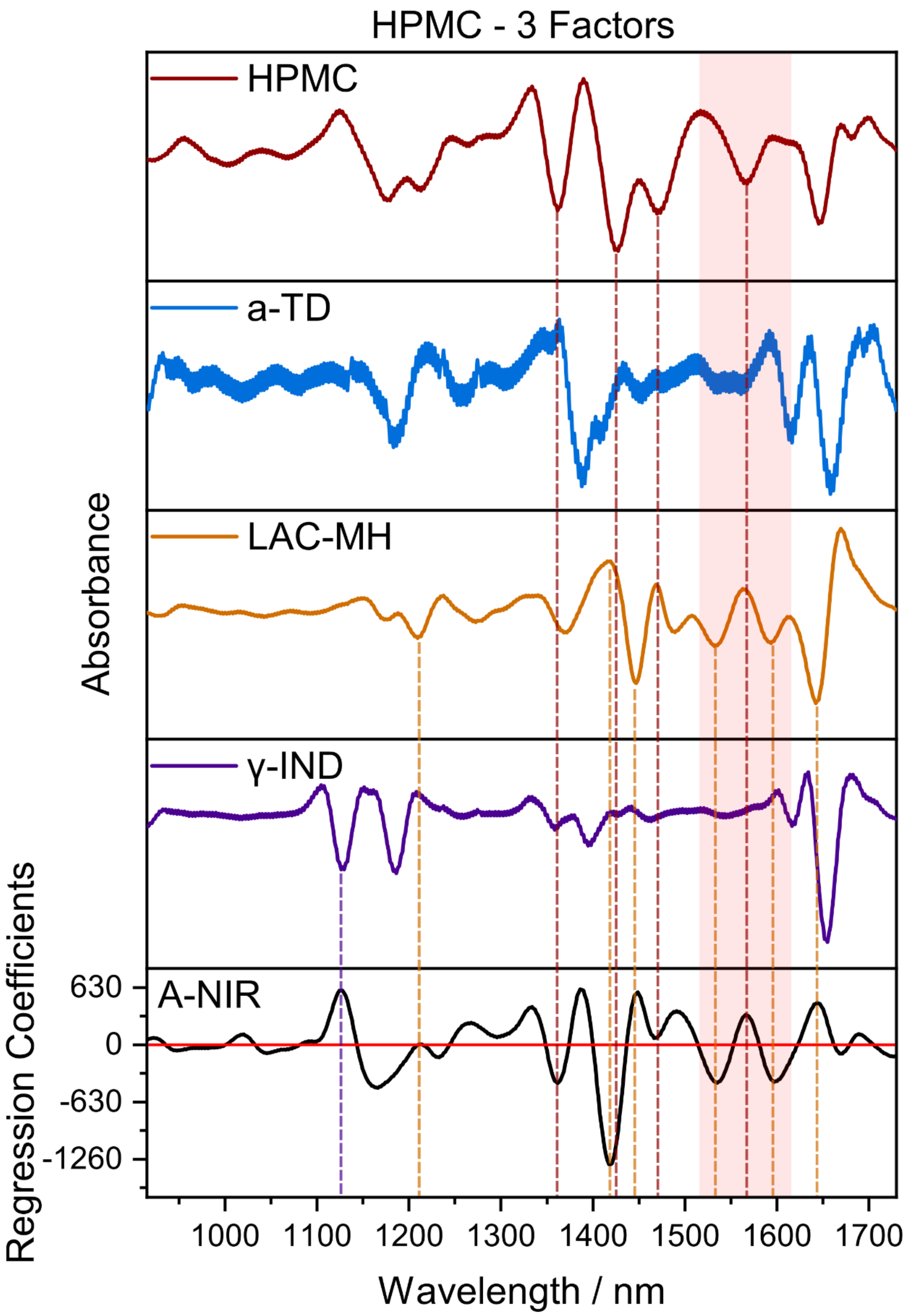

Figure S17. Reference preprocessed A-NIR spectra of KBr (teal), HPMC (red), a-TD (blue), LAC-MH (gold), and γ-IND (purple) compared against regression coefficients (3 factors) of A-NIR PLS model predicting for HPMC content in tablet samples.

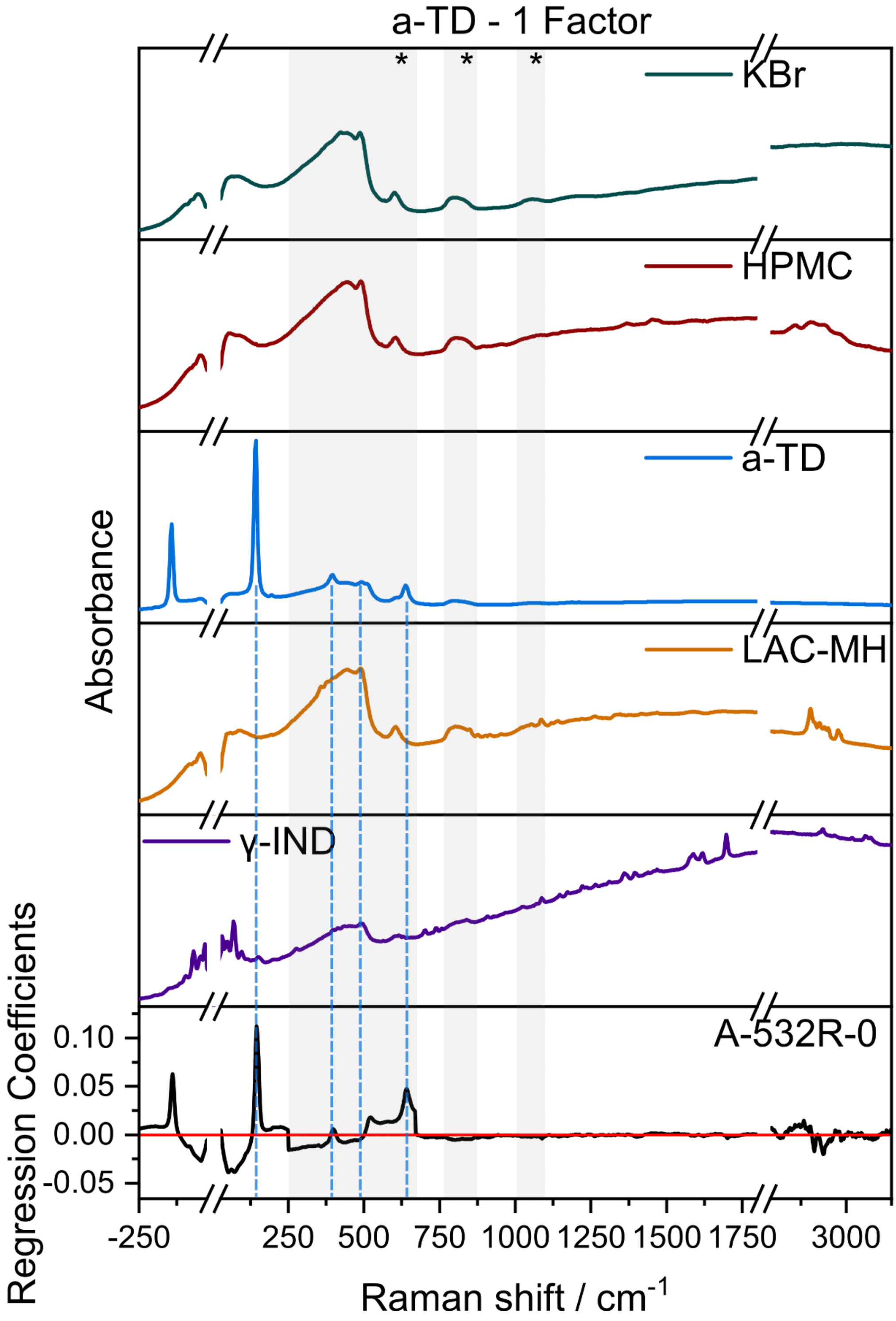

Figure S18. Reference preprocessed A-532R-0 spectra of KBr (teal), HPMC (red), a-TD (blue), LAC-MH (gold), and γ-IND (purple) compared against regression coefficients (1 factor) of A-532R-0 PLS model predicting for a-TD content in tablet samples.

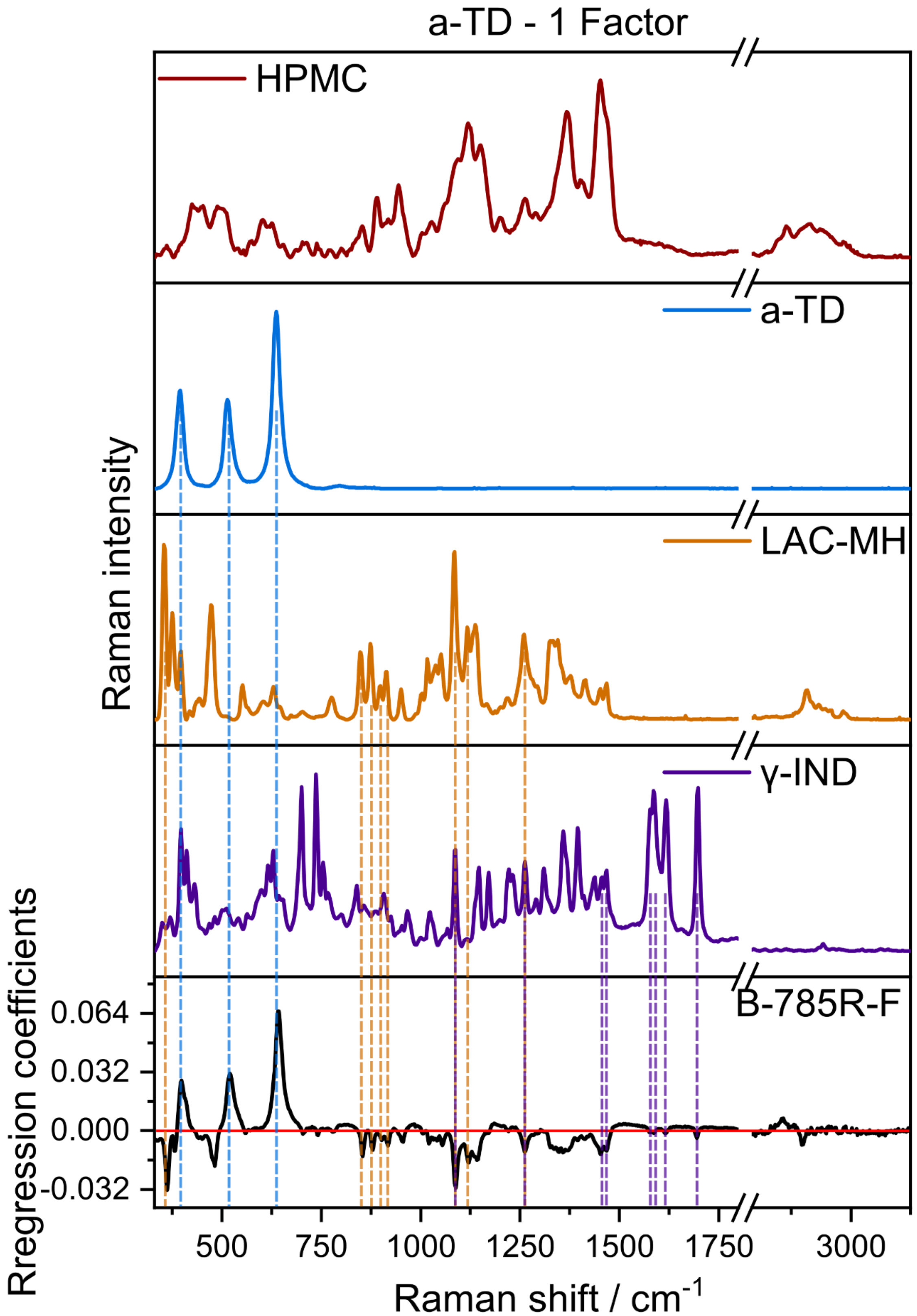

Figure S19. Reference preprocessed B-785R-F spectra of HPMC (red), a-TD (blue), LAC-MH (gold), and γ-IND (purple) compared against regression coefficients (1 factor) of A-B785R-F PLS model predicting for a-TD content in tablet samples.

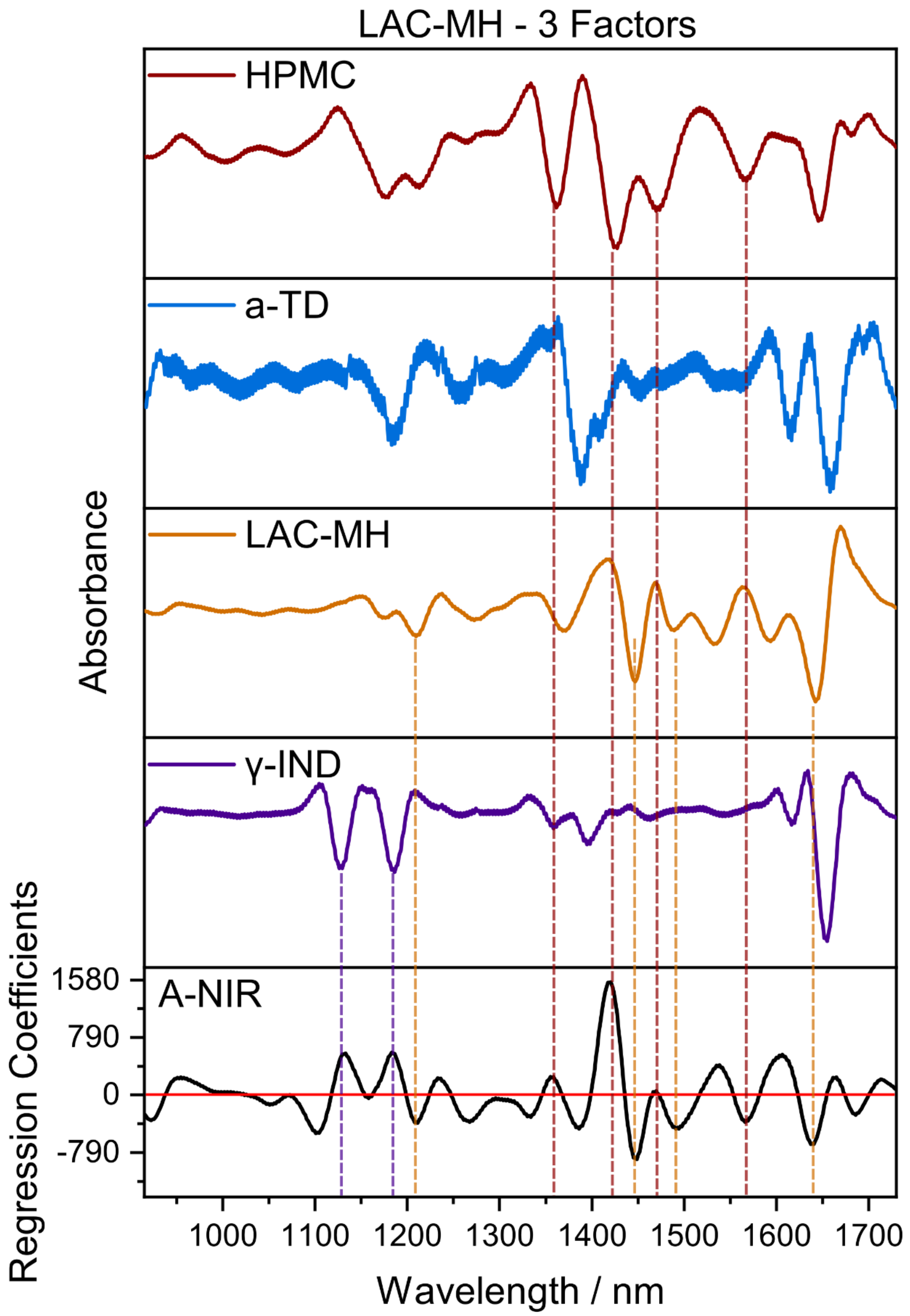

Figure S20. Reference preprocessed A-NIR spectra of KBr (teal), HPMC (red), a-TD (blue), LAC-MH (gold), and γ-IND (purple) compared against regression coefficients (3 factors) of A-NIR PLS model predicting for LAC-MH content in tablet samples.

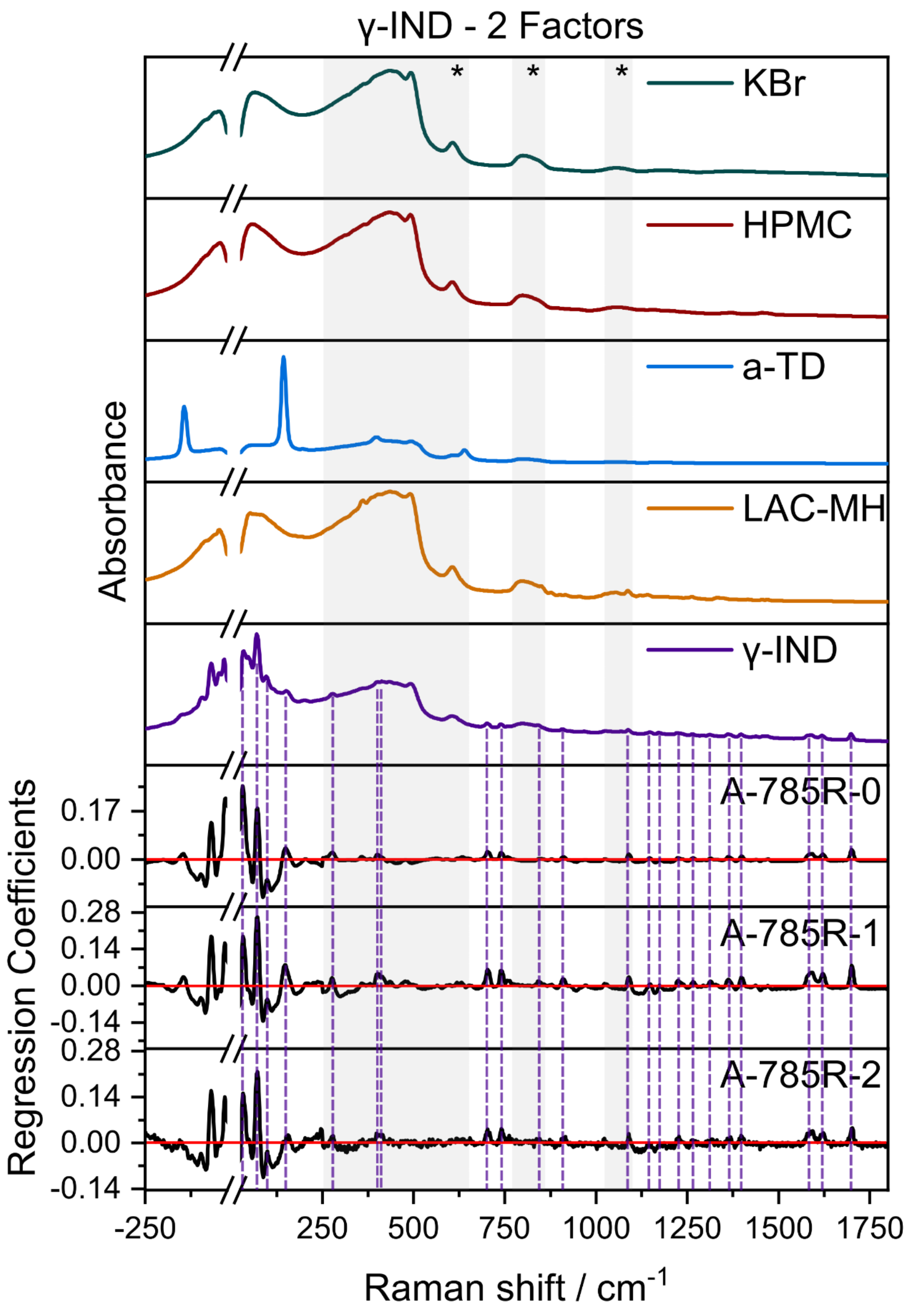

Figure S21. Reference preprocessed A-785R-0 spectra of KBr (teal), HPMC (red), a-TD (blue), LAC-MH (gold), and γ-IND (purple) compared against regression coefficients (2 factors) of A-785R-012 PLS model predicting for γ-IND content in tablet samples.

Table S2. Quantitative performance of predicting HPMC content in bilayer tablet samples. The PLS model was created for 12.8 to 43% w/w. Techniques with **bold** characters were the best valid performing technique within each wavelength. Techniques with ***italic and bold*** characters were the best performing technique overall.

	Technique	# of Factors	Calibration		Validation		Prediction	
			R_C^2	RMSEC / % w/w	R_V^2	RMSEV / % w/w	R_P^2	RMSEP / % w/w
Individual techniques	**A-532R-0**	**4**	**0.55**	**7.04**	**-0.55**	**13.13**	**0.33**	**5.58**
	A-532R-1	4	0.88	3.61	0.23	9.24	-0.30	7.77
	A-532R-2	1	0.40	8.18	-0.69	13.72	-0.11	7.16
	A-532B-0	5	0.18	9.51	-1.78	17.60	-0.06	7.01
	A-532B-1	1	0.04	10.32	-0.42	12.56	0.064	6.59
	**A-532B-2**	**2**	**0.20**	**9.41**	**-0.99**	**14.87**	**0.24**	**5.92**
	A-785R-0	***5***	***0.96***	***2.05***	***0.20***	***9.45***	***0.69***	***3.78***
	A-785R-1	3	0.92	3.00	0.77	5.09	0.37	5.40
	A-785R-2	3	0.89	3.52	0.20	9.41	0.66	3.97
	**A-NIR**	**6**	**0.94**	**2.61**	**0.05**	**10.30**	**0.11**	**6.43**
Fused techniques	**A-532R-012**	**4**	**0.93**	**2.71**	**-0.04**	**10.75**	**0.01**	**6.76**
	A-532R-0+NIR	3	0.78	4.91	-0.07	10.88	0.07	6.56
	A-532R-1+NIR	3	0.81	4.54	0.03	10.40	-0.08	7.07
	A-532R-2+NIR	4	0.95	2.36	-0.09	11.02	0.09	6.49
	**A-532R-012+NIR**	**4**	**0.95**	**2.39**	**-0.10**	**11.05**	**0.11**	**6.41**
	**A-785R-012**	**3**	**0.93**	**2.72**	**0.62**	**6.50**	**0.52**	**4.72**
	A-785R-0+NIR	3	0.75	5.27	-0.14	11.28	-0.05	6.97
	A-785R-1+NIR	3	0.84	4.21	0.11	9.96	0.11	6.41
	A-785R-2+NIR	4	0.91	3.18	0.29	8.88	0.07	6.56
	**A-785R-012+NIR**	**3**	**0.91**	**3.25**	**-0.01**	**10.59**	**0.22**	**6.00**
	A-532B-012	1	0.13	9.83	-0.48	12.82	-0.26	7.65
	A-532B-0+NIR	6	0.88	3.70	0.25	9.12	0.12	6.39
	A-532B-1+NIR	6	0.91	3.17	0.41	8.11	0.01	6.76
	A-532B-2+NIR	5	0.88	3.68	0.19	9.48	0.08	6.54
	A-532B-012+NIR	6	0.88	3.64	-0.12	11.16	0.08	6.52

Table S3. Quantitative performance of predicting a-TD content in bilayer tablet samples. The PLS model was created for 0 to 4.25% w/w. Techniques with **bold** characters were the best valid performing technique within each wavelength. Techniques with ***italic and bold*** characters were the best performing technique overall.

Technique	Calibration	Validation	Prediction

		# of Factors	R_C^2	RMSEC / % w/w	R_V^2	RMSEV / % w/w	R_P^2	RMSEP / % w/w
Individual techniques	**A-532R-0**	**1**	**0.85**	**0.68**	**0.79**	**0.82**	**0.96**	**0.28**
	**A-532R-1**	**1**	**0.87**	**0.62**	**0.81**	**0.78**	**0.96**	**0.28**
	A-532R-2	4	0.95	0.39	0.51	1.24	0.39	1.05
	**A-532B-0**	**4**	**0.63**	**1.08**	**0.21**	**1.58**	**0.60**	**0.85**
	A-532B-1	2	0.25	1.53	-0.65	2.28	0.39	1.04
	A-532B-2	3	0.48	1.28	0.01	1.77	0.56	0.89
	A-785R-0	***1***	***0.88***	***0.61***	***0.83***	***0.73***	***0.96***	***0.26***
	A-785R-1	1	0.90	0.56	0.85	0.68	0.94	0.31
	A-785R-2	1	0.88	0.60	0.83	0.73	0.95	0.30
	**A-NIR**	**2**	**0.89**	**0.60**	**0.67**	**1.02**	**0.81**	**0.58**
Fused techniques	**A-532R-012**	**1**	**0.87**	**0.63**	**0.80**	**0.80**	**0.96**	**0.27**
	A-532R-0+NIR	1	0.87	0.65	0.81	0.78	0.95	0.31
	A-532R-1+NIR	1	0.88	0.60	0.82	0.75	0.95	0.30
	A-532R-2+NIR	3	0.94	0.43	0.67	1.02	0.60	0.85
	A-532R-012+NIR	1	0.88	0.62	0.81	0.78	0.95	0.29
	**A-785R-012**	**1**	**0.89**	**0.58**	**0.84**	**0.70**	**0.95**	**0.29**
	A-785R-0+NIR	1	0.89	0.58	0.85	0.70	0.95	0.30
	A-785R-1+NIR	1	0.91	0.55	0.87	0.65	0.94	0.33
	A-785R-2+NIR	1	0.90	0.57	0.85	0.68	0.94	0.33
	A-785R-012+NIR	1	0.89	0.57	0.85	0.69	0.94	0.30
	A-532B-012	3	0.65	1.06	0.05	1.73	0.34	1.09
	A-532B-0+NIR	4	0.90	0.57	0.58	1.15	0.74	0.68
	A-532B-1+NIR	2	0.80	079	0.55	1.19	0.66	0.78
	**A-532B-2+NIR**	**2**	**0.88**	**0.61**	**0.68**	**1.01**	**0.76**	**0.65**
	A-532B-012+NIR	3	0.87	0.64	0.56	1.18	0.60	0.85

Table S4. Quantitative performance of predicting LAC-MH content in bilayer tablet samples. The PLS model was created for 45.45 to 87.46% w/w. Techniques with **bold** characters were the best valid performing technique within each wavelength. Techniques with ***italic and bold*** characters were the best performing technique overall.

	Technique	# of Factors	Calibration		Validation		Prediction	
			R_C^2	RMSEC / % w/w	R_V^2	RMSEV / % w/w	R_P^2	RMSEP / % w/w
Individual techniques	***A-532R-0***	***4***	***0.58***	***8.05***	***-0.41***	***14.80***	***0.65***	***5.75***
	A-532R-1	4	0.91	3.78	0.23	10.92	0.04	9.49
	A-532R-2	1	0.47	9.07	0.01	12.40	0.57	6.32
	A-532B-0	3	0.27	10.65	-0.62	15.85	-0.03	9.83
	A-532B-1	3	0.26	10.73	-1.14	18.22	0.13	9.01
	**A-532B-2**	**3**	**0.56**	**8.28**	**0.10**	**11.80**	**0.64**	**5.78**
	A-785R-0	5	0.88	4.39	-0.28	14.11	0.39	7.57
	A-785R-1	2	0.66	7.24	-0.13	13.27	0.38	7.65

	**A-785R-2**	**2**	**0.50**	**8.82**	**-0.77**	**16.55**	**0.48**	**6.96**
	**A-NIR**	**4**	**0.79**	**5.67**	**-0.28**	**14.07**	**0.15**	**8.90**
Fused techniques	**A-532R-012**	**2**	**0.64**	**7.48**	**-0.02**	**12.55**	**0.54**	**6.54**
	A-532R-0+NIR	6	0.90	3.89	0.09	11.89	0.13	9.03
	A-532R-1+NIR	5	0.96	2.41	0.11	11.75	-0.02	9.76
	A-532R-2+NIR	5	0.98	1.88	0.25	10.80	0.46	7.11
	A-532R-012+NIR	2	0.70	6.79	-0.03	12.68	0.48	6.99
	**A-785R-012**	**2**	**0.62**	**7.67**	**-0.40**	**14.74**	**0.46**	**7.09**
	A-785R-0+NIR	4	0.73	6.43	-0.22	13.74	-0.31	11.06
	A-785R-1+NIR	5	0.86	4.74	-0.11	13.13	0.10	9.20
	A-785R-2+NIR	5	0.91	3.68	0.01	12.35	0.13	9.05
	A-785R-012+NIR	5	0.94	2.98	0.01	12.45	0.02	9.56
	A-532B-012	2	0.33	10.20	-0.54	15.47	0.01	9.67
	A-532B-0+NIR	5	0.82	5.22	-0.90	17.18	0.09	9.24
	A-532B-1+NIR	5	0.89	4.09	0.08	11.94	0.12	9.10
	A-532B-2+NIR	4	0.89	4.21	0.34	10.10	0.30	8.11
	A-532B-012+NIR	6	0.91	3.80	-0.08	12.95	0.18	8.77

Table S5. Quantitative performance of predicting γ-IND content in bilayer tablet samples. The PLS model was created for 0to 17.72% w/w. Techniques with **bold** characters were the best valid performing technique within each wavelength. Techniques with ***italic and bold*** characters were the best performing technique overall.

	Technique	# of Factors	Calibration		Validation		Prediction	
			R_C^2	RMSEC / % w/w	R_V^2	RMSEV / % w/w	R_P^2	RMSEP / % w/w
ndividual techniques	A-532R-0	4	0.53	4.98	-0.20	7.93	0.36	5.90
	A-532R-1	4	0.86	2.68	-0.64	9.28	0.54	5.00
	**A-532R-2**	**1**	**0.63**	**4.43**	**0.44**	**5.43**	**0.66**	**4.26**
	A-532B-0	2	0.25	6.27	-0.77	9.63	0.34	5.96
	A-532B-1	2	0.61	4.51	0.44	5.43	0.49	5.26
	A-532B-2	**2**	***0.92***	***1.99***	***0.78***	***3.41***	***0.89***	***2.48***
	A-785R-0	3	0.40	5.59	-0.64	9.25	0.38	5.76
	A-785R-1	2	0.53	4.95	0.20	6.46	0.58	4.79
	**A-785R-2**	**2**	**0.59**	**4.61**	**0.31**	**6.00**	**0.61**	**4.59**
	**A-NIR**	**4**	**0.53**	**4.98**	**-6.70**	**20.07**	**0.21**	**6.53**
Fused techniques	A-532R-012	2	0.72	3.85	0.33	5.94	0.70	4.01
	A-532R-0+NIR	2	0.31	6.01	-0.43	8.65	0.20	6.56
	A-532R-1+NIR	2	0.51	5.08	-0.53	8.97	0.13	6.87
	A-532R-2+NIR	1	0.69	4.02	0.43	5.44	0.68	4.13
	**A-532R-012+NIR**	**2**	**0.75**	**3.63**	**0.29**	**6.11**	**0.71**	**3.96**
	**A-785R-012**	**2**	**0.58**	**4.68**	**0.27**	**6.18**	**0.60**	**4.66**
	A-785R-0+NIR	1	0.18	6.57	-0.93	10.05	0.06	7.13
	A-785R-1+NIR	2	0.44	5.40	-0.18	7.84	0.32	6.06

A-785R-2+NIR	2	0.49	5.18	-0.08	7.51	0.36	5.86
A-785R-012+NIR	2	0.56	4.78	0.01	7.20	0.46	5.42
A-532B-012	2	0.67	4.14	0.53	4.98	0.61	4.62
A-532B-0+NIR	1	0.23	6.36	-0.60	9.16	0.20	6.56
A-532B-1+NIR	1	0.53	4.94	0.27	6.18	0.41	5.64
A-532B-2+NIR	2	0.76	3.58	0.30	6.07	0.60	4.65
**A-532B-012+NIR**	**2**	**0.65**	**4.30**	**0.34**	**5.86**	**0.61**	**4.61**

Table S6. Quantitative performance of predicting thickness of bilayer tablet samples. The PLS model was created for 3.45 to 5.55 mm. Techniques with **bold** characters were the best valid performing technique within each wavelength. Techniques with ***italic and bold*** characters were the best performing technique overall.

	Technique	# of Factors	Calibration		Validation		Prediction	
			R_C^2	RMSEC / mm	R_V^2	RMSEV / mm	R_P^2	RMSEP / mm
Individual techniques	A-532R-0	4	0.49	0.57	-0.85	1.09	-0.19	0.51
	A-532R-1	1	0.11	0.75	-0.21	0.8	-0.51	0.58
	A-532R-2	2	0.71	0.43	-0.23	0.89	-0.51	0.58
	A-532B-0	1	0.14	0.74	-0.22	0.89	-0.67	0.61
	A-532B-1	1	0.03	0.79	-0.47	0.97	-0.19	0.51
	A-532B-2	2	0.21	0.71	-0.42	0.95	-0.02	0.48
	A-785R-0	5	0.95	0.17	-0.18	0.87	0.19	0.42
	A-785R-1	3	0.86	0.30	0.70	0.44	-0.22	0.52
	A-785R-2	***3***	***0.86***	***0.30***	***0.21***	***0.71***	***0.31***	***0.39***
	**A-NIR**	1	0.28	0.68	-0.40	0.95	-0.88	0.65
Fused techniques	**A-532R-012**	1	0.11	0.75	-0.25	0.90	-0.50	0.58
	A-532R-0+NIR	**3**	0.75	0.40	-0.09	0.84	-0.64	0.60
	A-532R-1+NIR	3	0.79	0.37	-0.04	0.82	-0.84	0.64
	A-532R-2+NIR	4	0.95	0.18	-0.15	0.86	-0.56	0.59
	**A-532R-012+NIR**	4	0.94	0.20	-0.19	0.87	-0.61	0.60
	**A-785R-012**	**3**	**0.89**	**0.27**	**0.59**	**0.51**	**0.01**	**0.47**
	A-785R-0+NIR	3	0.71	0.43	-0.18	0.87	-0.73	0.62
	A-785R-1+NIR	3	0.80	0.36	0.10	0.76	-0.61	0.60
	A-785R-2+NIR	3	0.77	0.38	-0.12	0.85	-0.66	0.61
	A-785R-012+NIR	3	0.85	0.31	0.02	0.79	-0.54	0.58
	A-532B-012	1	0.15	0.74	-0.32	0.92	-0.62	0.60
	A-532B-0+NIR	5	0.80	0.36	0.11	0.76	-0.75	0.62
	A-532B-1+NIR	4	0.80	0.36	0.12	0.75	-0.94	0.66
	A-532B-2+NIR	4	0.80	0.36	-0.06	0.83	-0.84	0.64
	A-532B-012+NIR	5	0.81	0.34	-0.43	0.96	-0.79	0.63

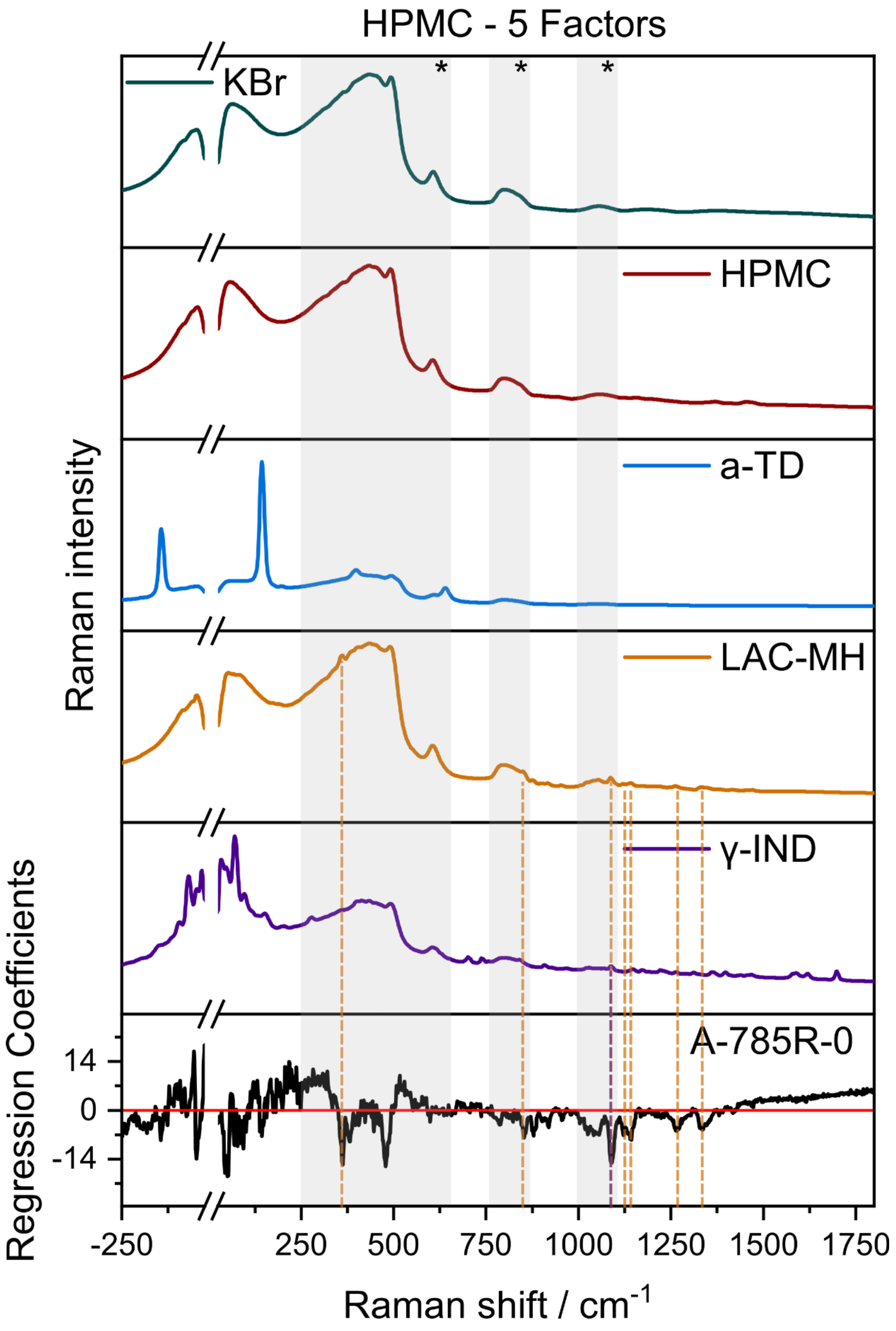

Figure S22. Reference preprocessed A-785R-0 spectra of KBr (teal), HPMC (red), a-TD (blue), LAC-MH (gold), and γ-IND (purple) compared against regression coefficients (2 factors) of A-785R-0 PLS model for predicting HPMC content in bilayers.

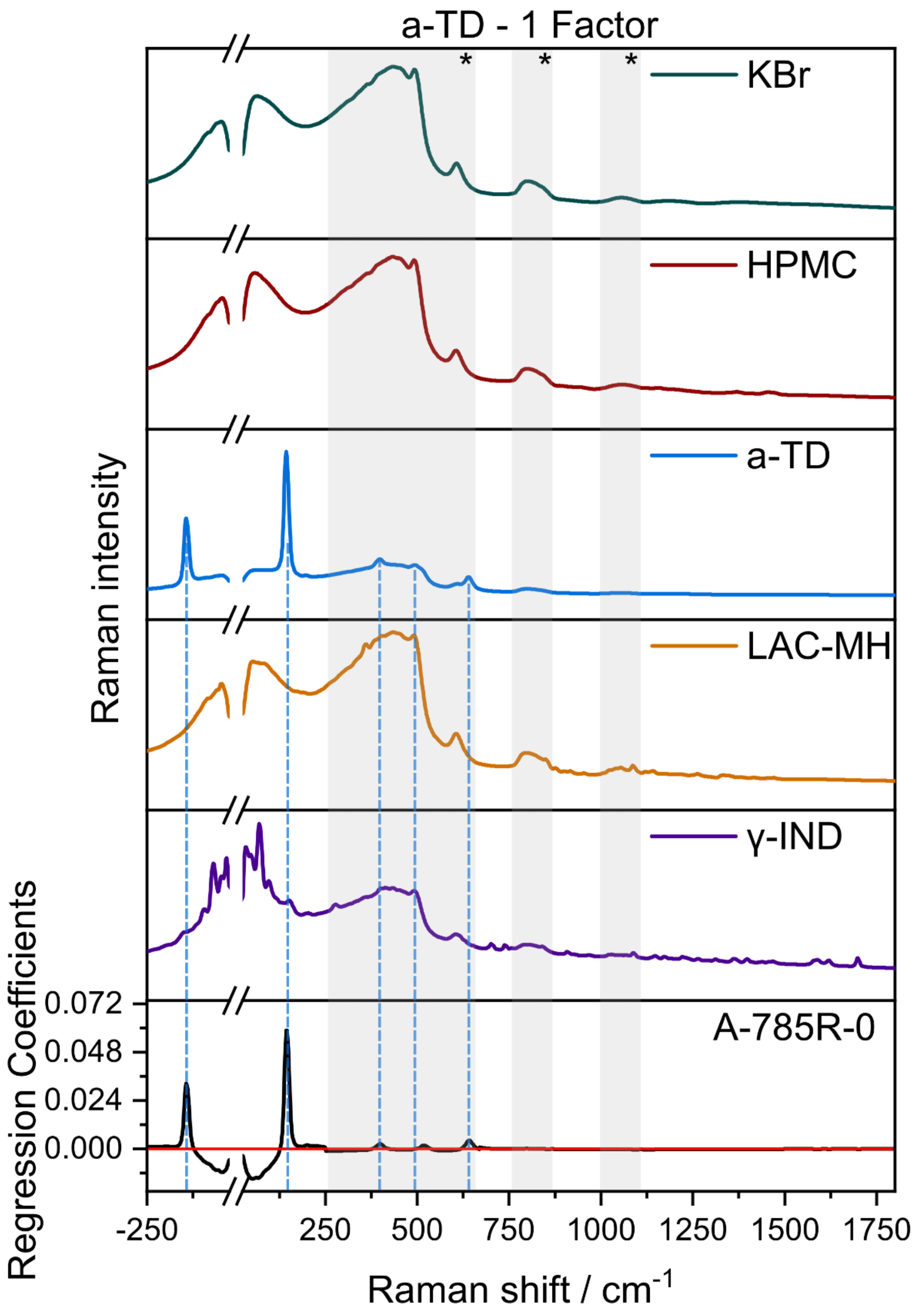

Figure S23. Reference preprocessed A-785R-0 spectra of KBr (teal), HPMC (red), a-TD (blue), LAC-MH (gold), and γ-IND (purple) compared against regression coefficients (2 factors) of A-785R-0 PLS model for predicting a-TD content in bilayers.

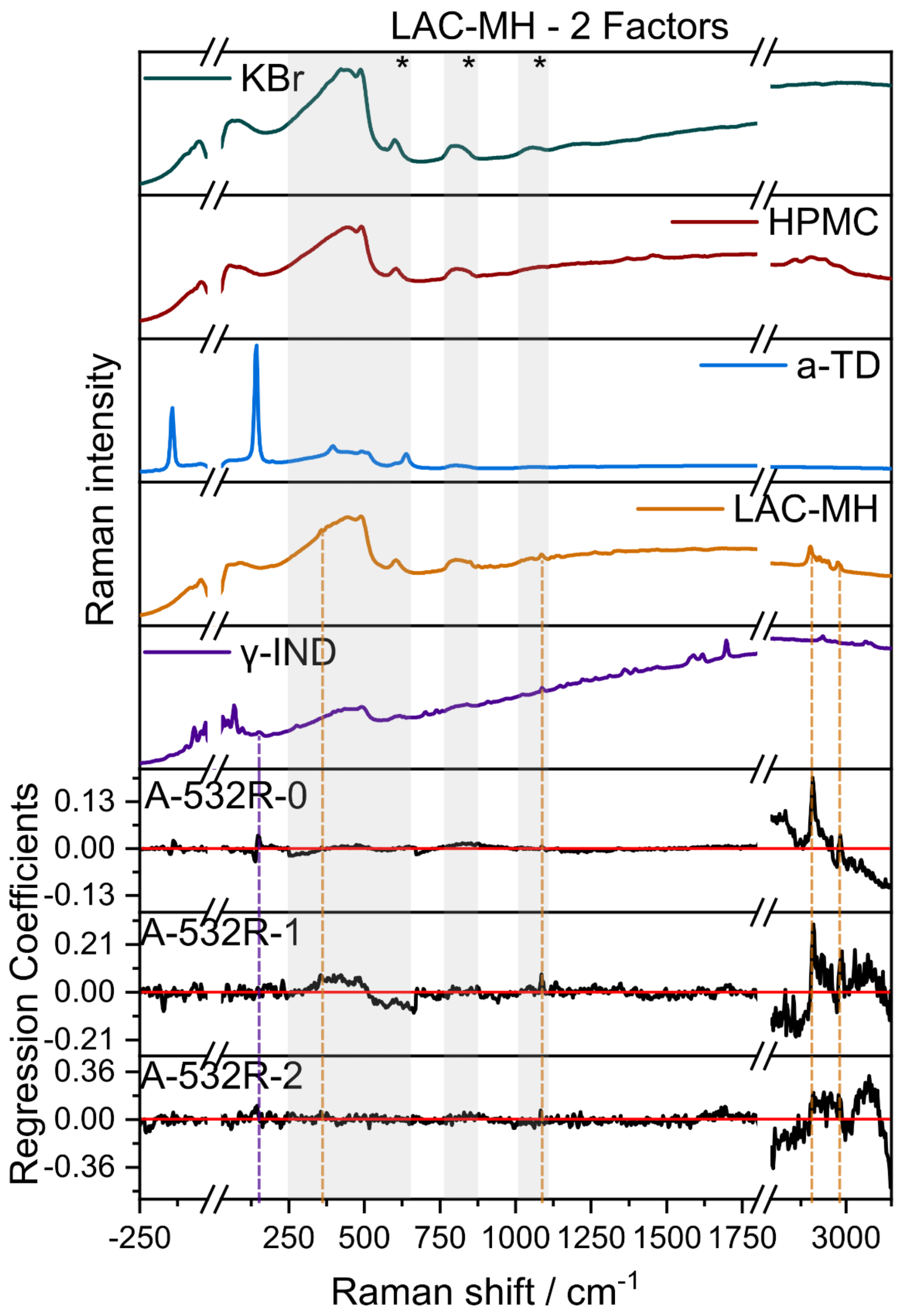

Figure S24. Reference preprocessed A-532R-0 spectra of KBr (teal), HPMC (red), a-TD (blue), LAC-MH (gold), and γ-IND (purple) compared against regression coefficients (2 factors) of A-532R-012 PLS model for predicting LAC-MH content in bilayers.

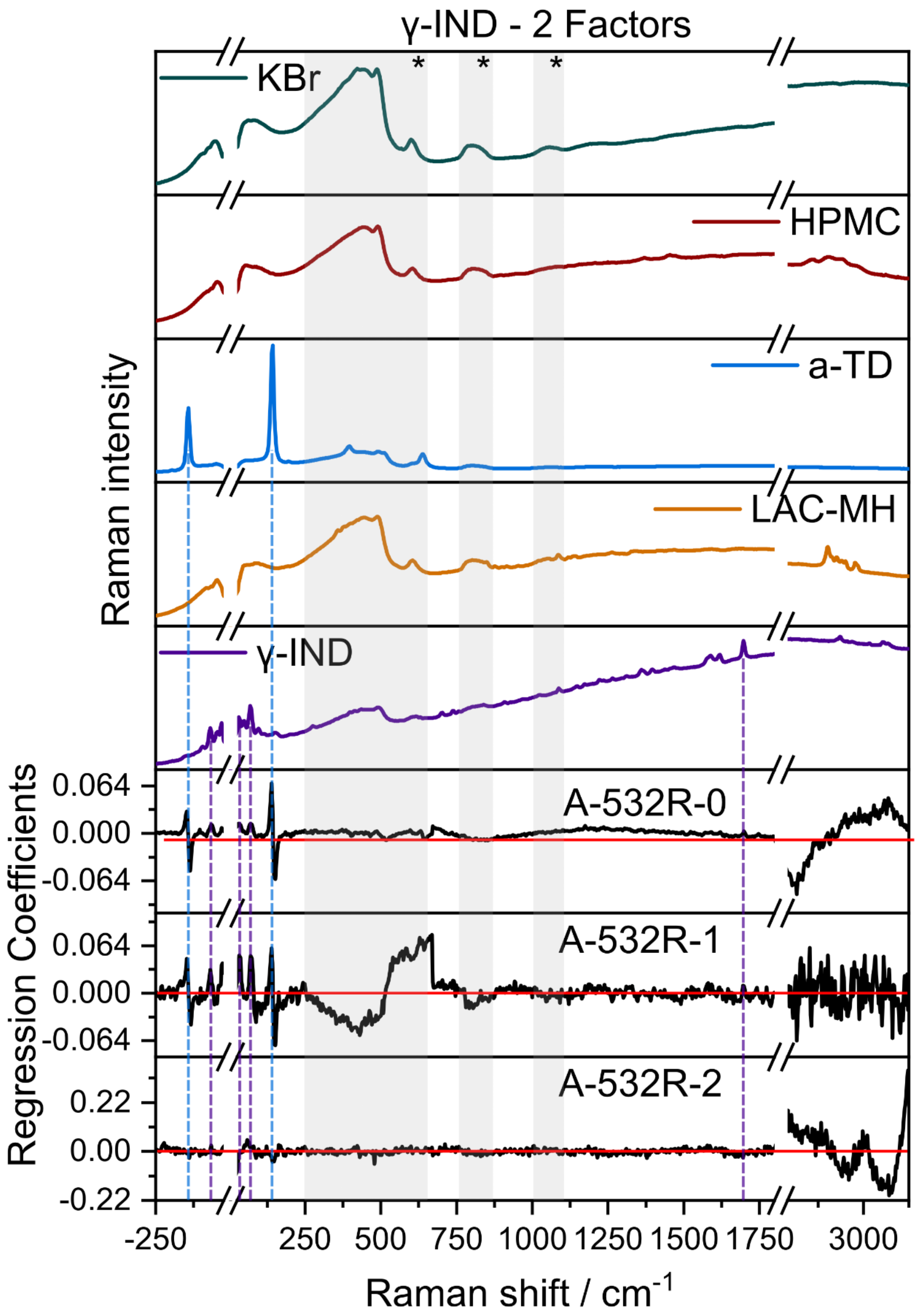

Figure S25. Reference preprocessed A-532R-0 spectra of KBr (teal), HPMC (red), a-TD (blue), LAC-MH (gold), and γ-IND (purple) compared against regression coefficients (2 factors) of A-532R-012 PLS model for predicting γ-IND content in bilayers.

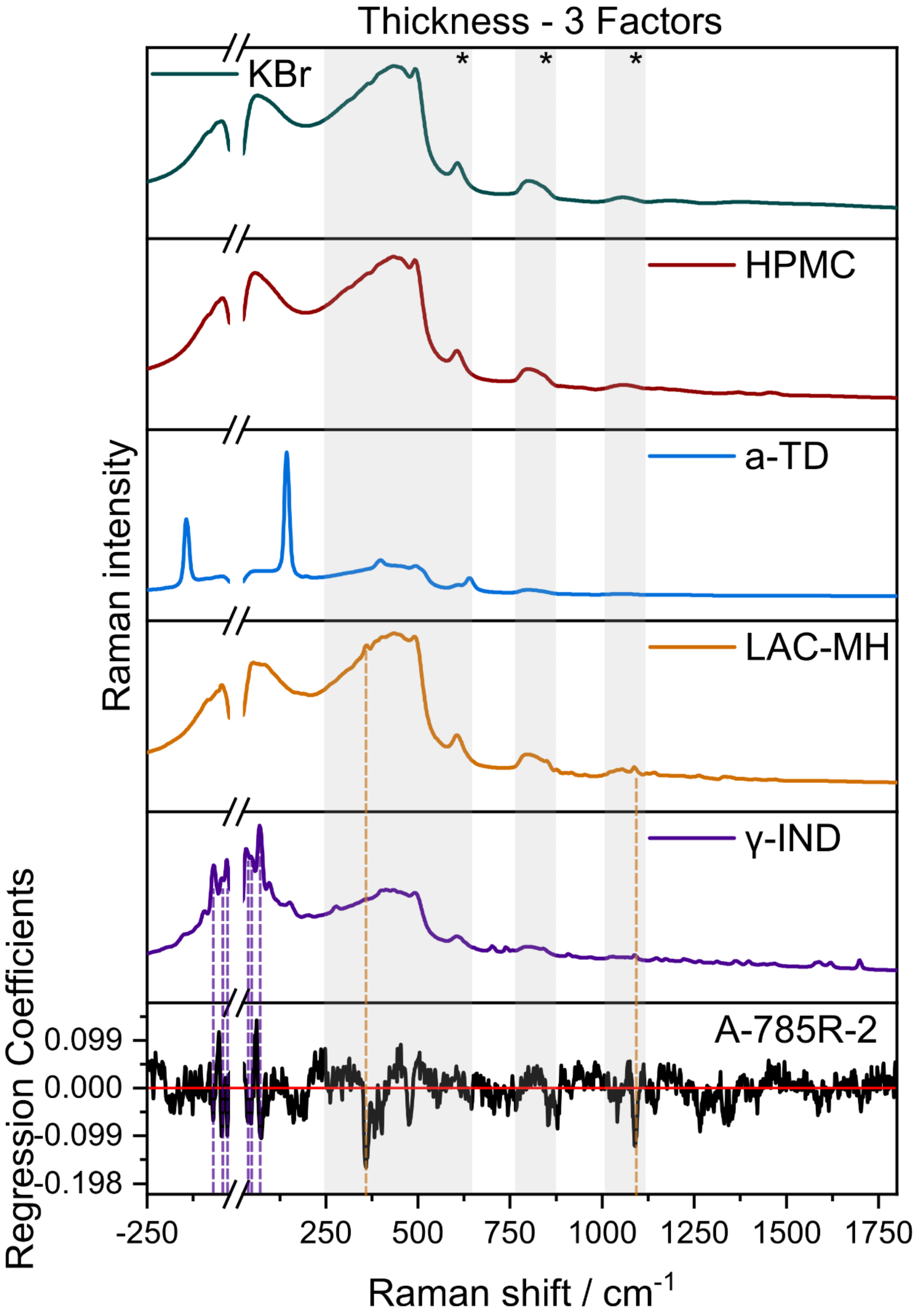

Figure S26. Reference preprocessed A-785R-0 spectra of KBr (teal), HPMC (red), a-TD (blue),

LAC-MH (gold), and γ-IND (purple) compared against regression coefficients (2 factors) of A-785R-2 PLS model for predicting bilayer thickness.

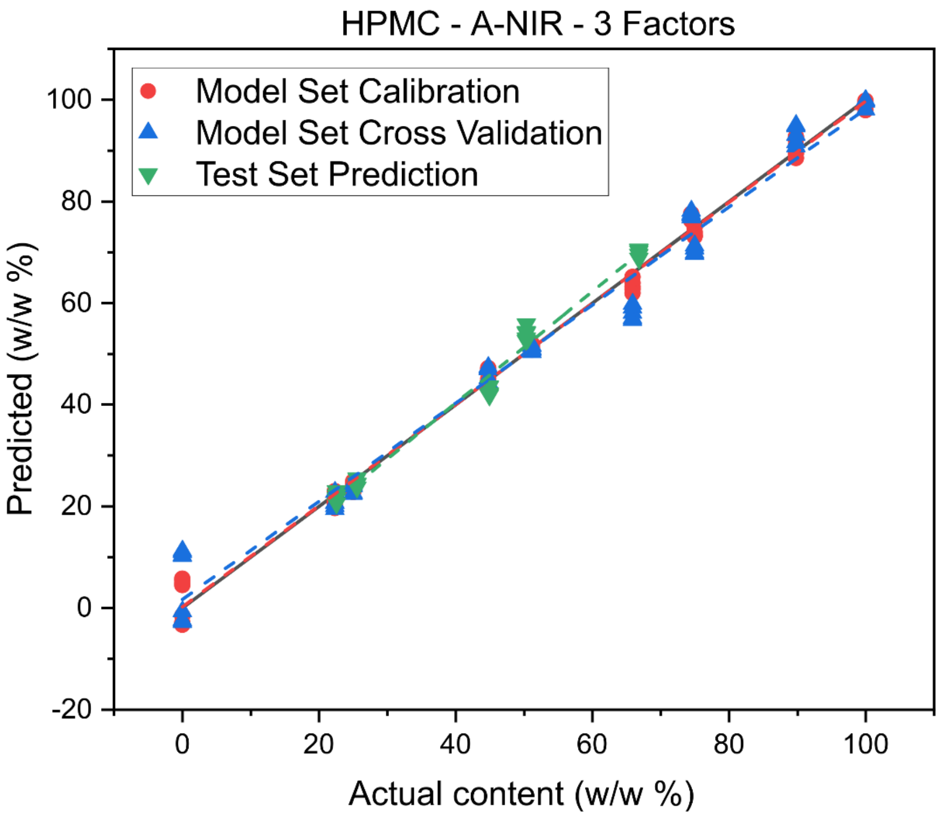

Figure S27. Plot of actual vs prediction HPMC content in tablet samples using PLS model developed with A-NIR data with 3 factors.

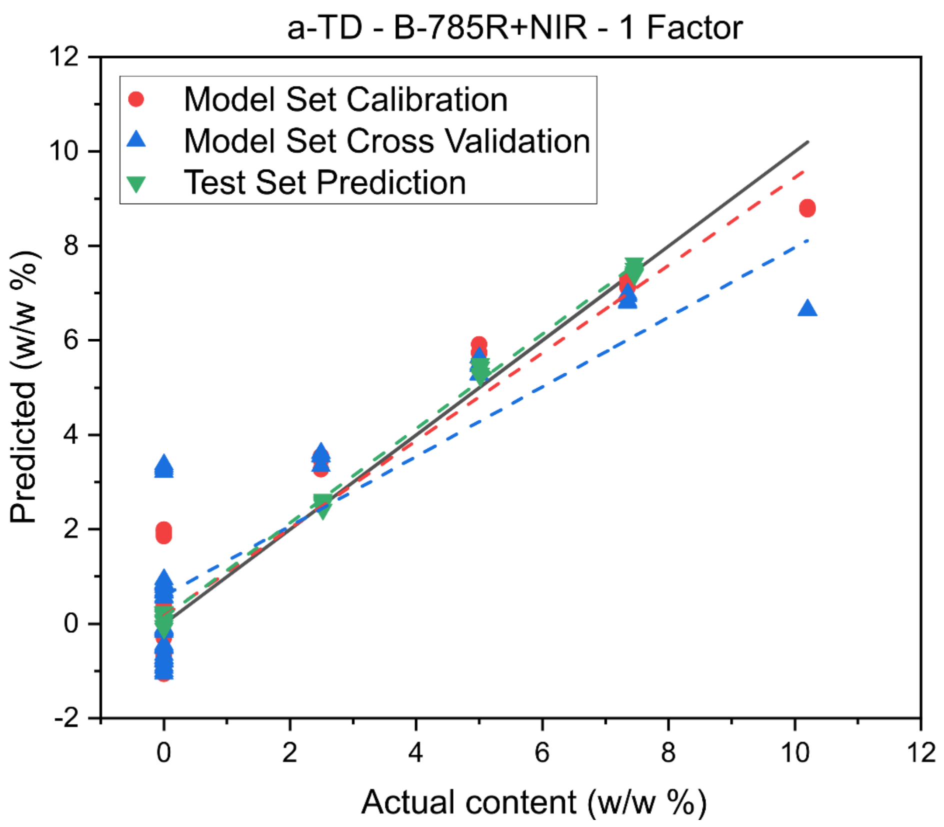

Figure S28. Plot of actual vs prediction a-TD content in tablet samples using PLS model developed with B-785R+NIR data with 1 factor.

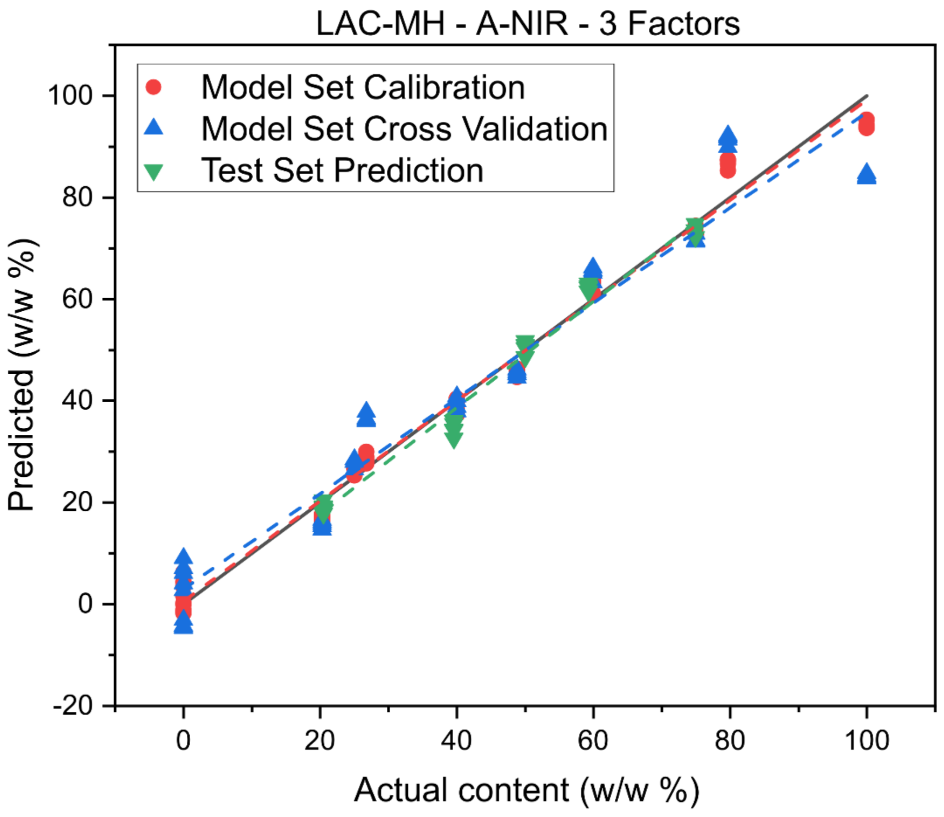

Figure S29. Plot of actual vs prediction LAC-MH content in tablet samples using PLS model developed with A-NIR data with 3 factors.

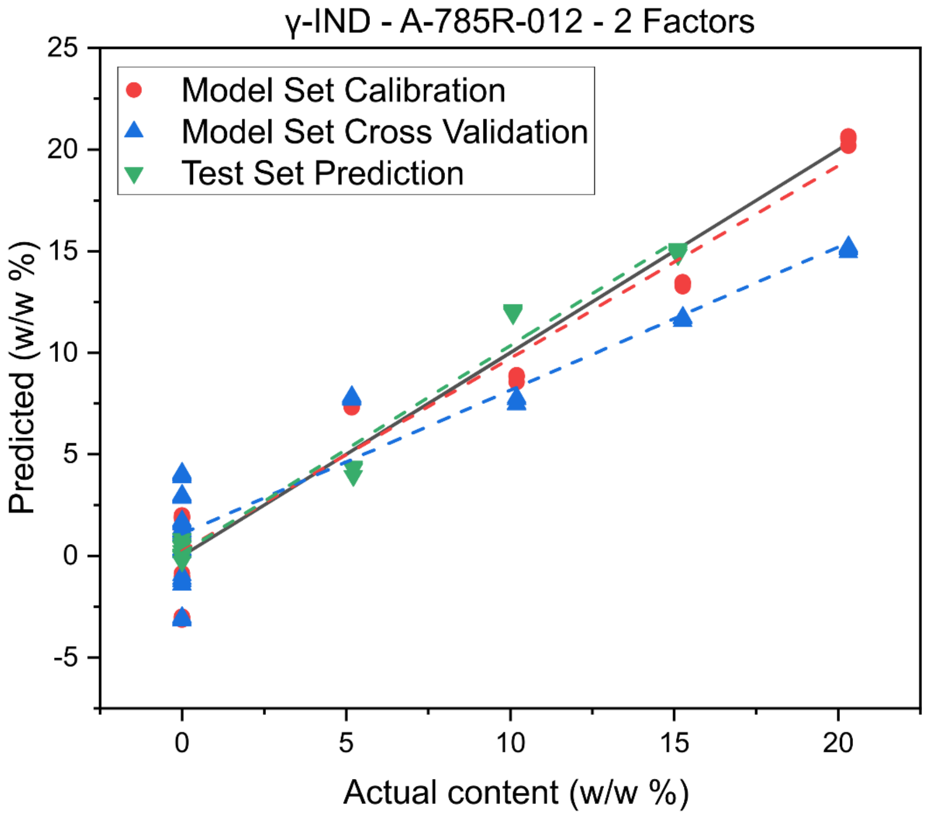

Figure S30. Plot of actual vs prediction γ-IND content in tablet samples using PLS model developed with A- data with 2 factors.

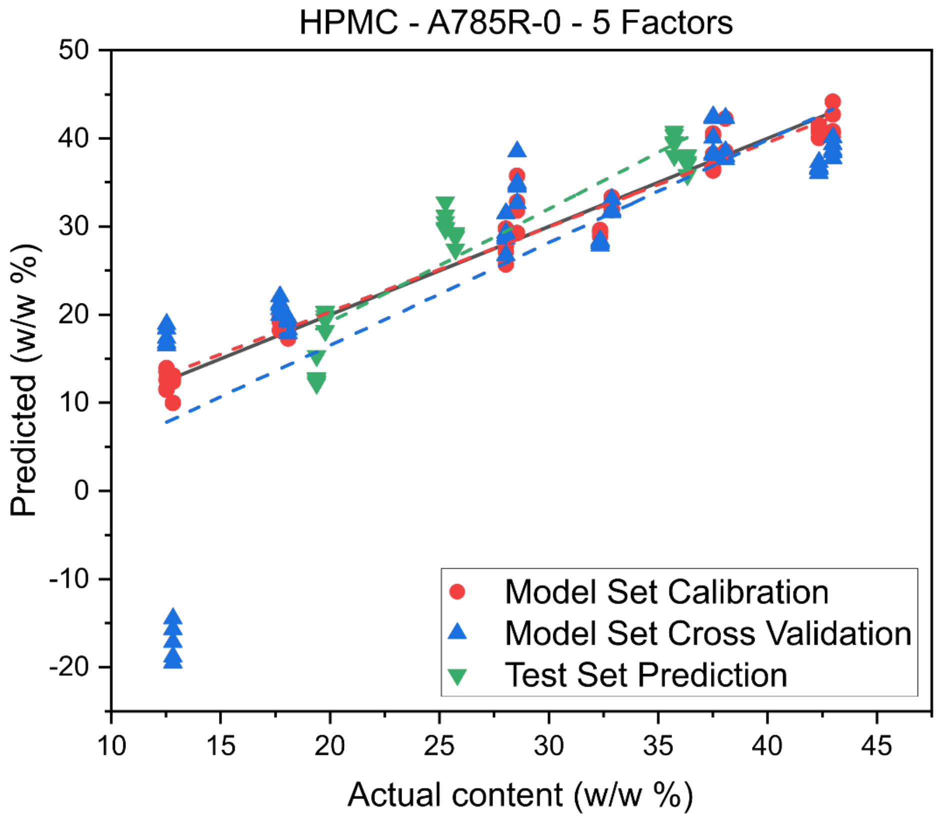

Figure S31. Plot of actual vs prediction HPMC content in BL samples using PLS model developed with A-785R-0 data with 5 factors.

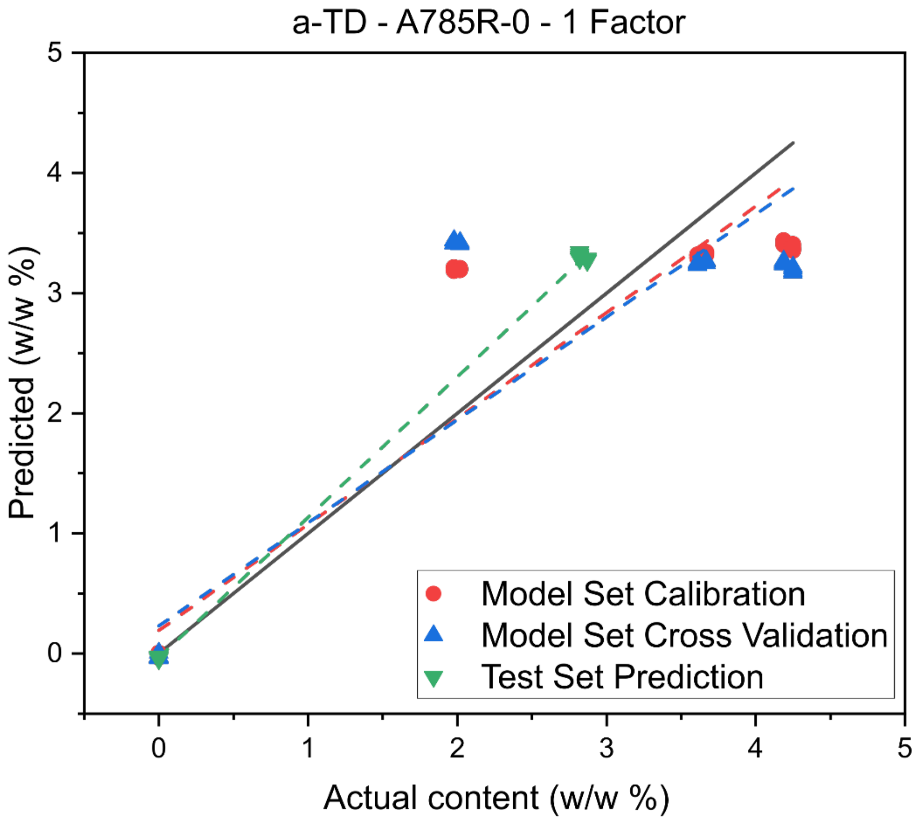

Figure S32. Plot of actual vs prediction a-TD content in BL samples using PLS model developed with A-785R-0 data with 1 factor.

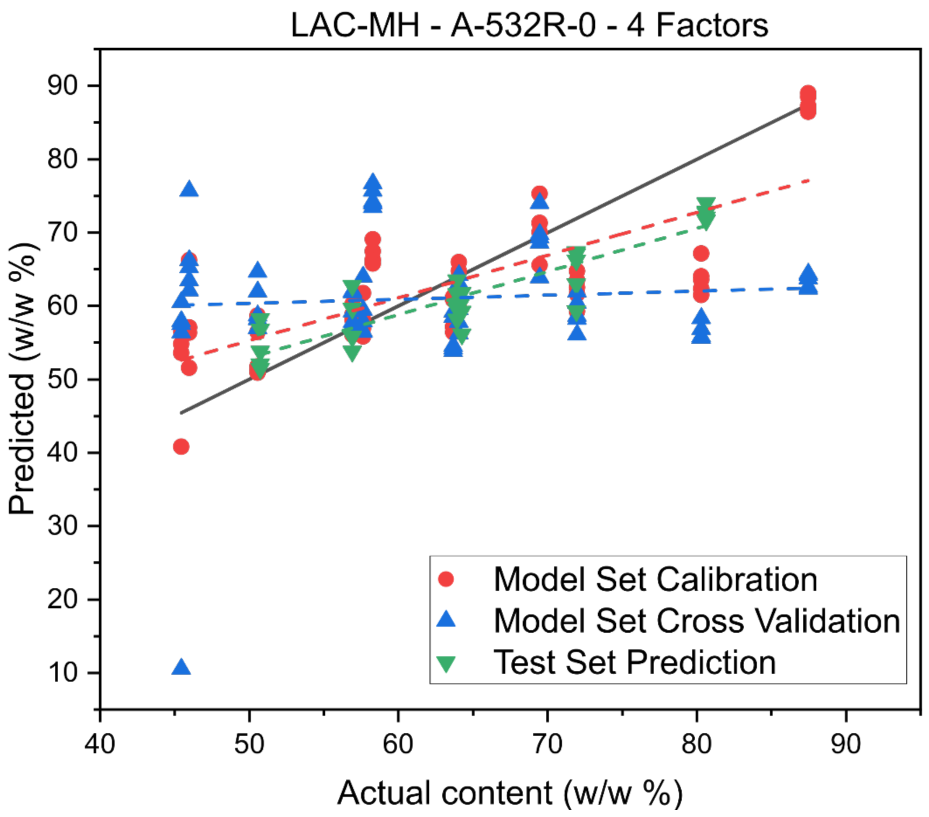

Figure S33. Plot of actual vs prediction LAC-MH content in BL samples using PLS model developed with A-532R-0 data with 4 factors.

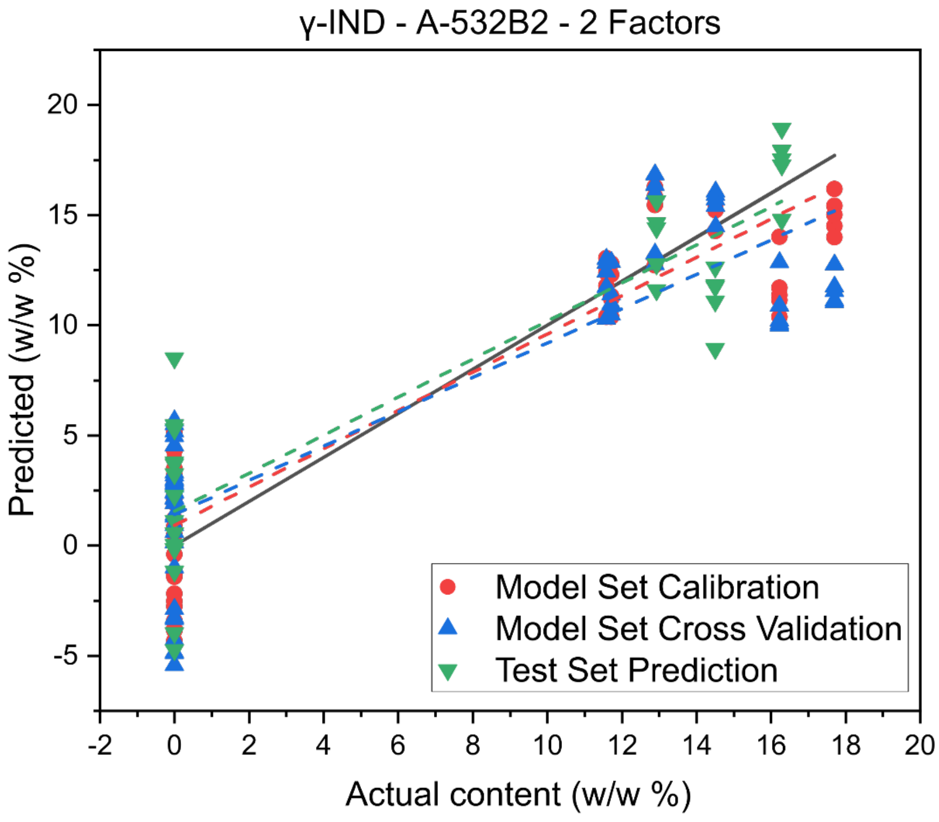

Figure S34. Plot of actual vs prediction γ-IND content in BL samples using PLS model developed with A-532B-2 data with 2 factors.

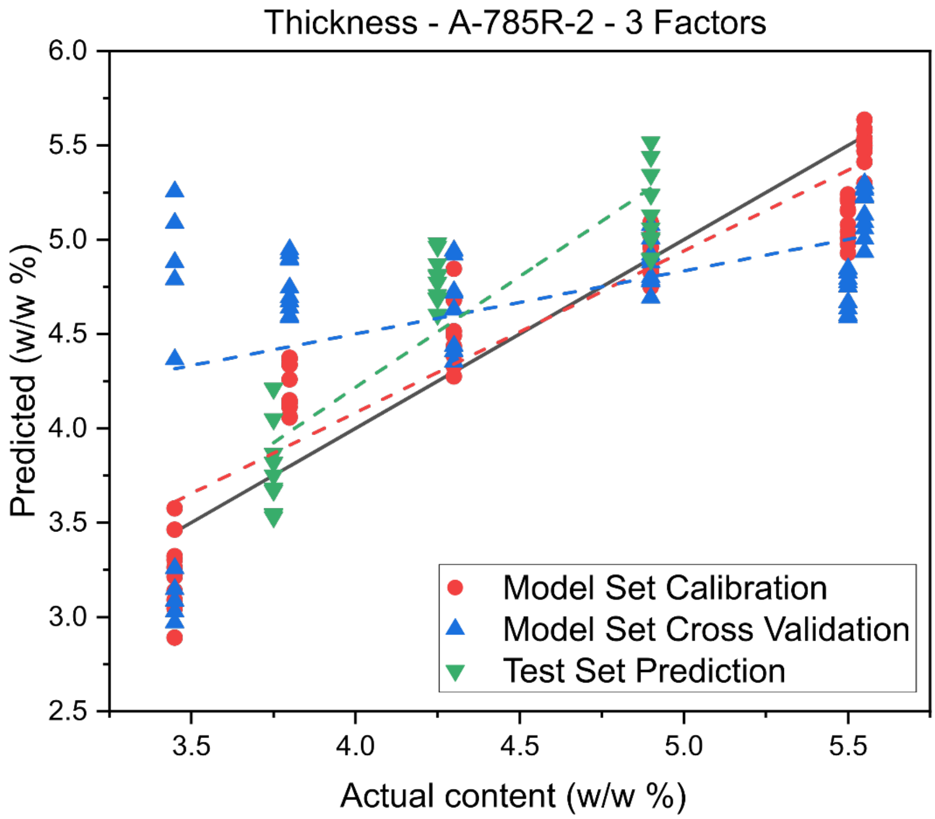

Figure S35. Plot of actual vs prediction thickness of BL samples using PLS model developed with A-785R-2 data with 3 factors.
